# Probing Anti-Proliferative 24-Homoscalaranes from a Sponge *Lendenfeldia* sp.

**DOI:** 10.3390/md18020076

**Published:** 2020-01-24

**Authors:** Bo-Rong Peng, Kuei-Hung Lai, You-Ying Chen, Jui-Hsin Su, Yusheng M. Huang, Yu-Hsin Chen, Mei-Chin Lu, Steve Sheng-Fa Yu, Chang-Yih Duh, Ping-Jyun Sung

**Affiliations:** 1 Doctoral Degree Program in Marine Biotechnology, National Sun Yat-sen University, Kaohsiung 80424, Taiwan; pengpojung@gmail.com; 2 Doctoral Degree Program in Marine Biotechnology, Academia Sinica, Taipei 11529, Taiwan; 3 National Museum of Marine Biology and Aquarium, Pingtung 94450, Taiwan; zoeblack0108@gmail.com (Y.-Y.C.); x2219@nmmba.gov.tw (J.-H.S.); kb5634@yahoo.com.tw (Y.-H.C.); jinx6609@nmmba.gov.tw (M.-C.L.); 4 Research Center for Chinese Herbal Medicine, College of Human Ecology, Chang Gung University of Science and Technology, Taoyuan 33303, Taiwan; mos19880822@gmail.com; 5 Department of Marine Biotechnology and Resources, National Sun Yat-sen University, Kaohsiung 80424, Taiwan; 6 Graduate Institute of Marine Biology, National Dong Hwa University, Pingtung 94450, Taiwan; 7 Department of Marine Recreation, National Penghu University of Science and Technology, Penghu 88046, Taiwan; yusheng@npu.edu.tw; 8 Institute of Chemistry, Academia Sinica, Taipei 11529, Taiwan; 9 Chinese Medicine Research and Development Center, China Medical University Hospital, Taichung 40447, Taiwan; 10 Graduate Institute of Natural Products, Kaohsiung Medical University, Kaohsiung 80708, Taiwan

**Keywords:** scalarane, sesterterpenoid, anti-proliferation, *Lendenfeldia*

## Abstract

In the current study, an NMR spectroscopic pattern-based procedure for probing scalarane derivatives was performed and four new 24-homoscalaranes, lendenfeldaranes A–D (**1**– **4**), along with three known compounds, 12α-acetoxy-22-hydroxy-24-methyl-24-oxoscalar-16-en- 25-al (**5**), felixin F (**6**), and 24-methyl-12,24,25-trioxoscalar-16-en-22-oic acid (**7**) were isolated from the sponge *Lendenfeldia* sp. The structures of scalaranes **1**–**7** were elucidated on the basis of spectroscopic analysis. Scalaranes **1**–**7** were further evaluated for their cytotoxicity toward a series of human cancer cell lines and the results suggested that **5** and **7** dominated in the anti- proliferative activity of the extract. The 18-aldehyde functionality was found to play a key role in their activity.

## 1. Introduction

**Figure 1:**
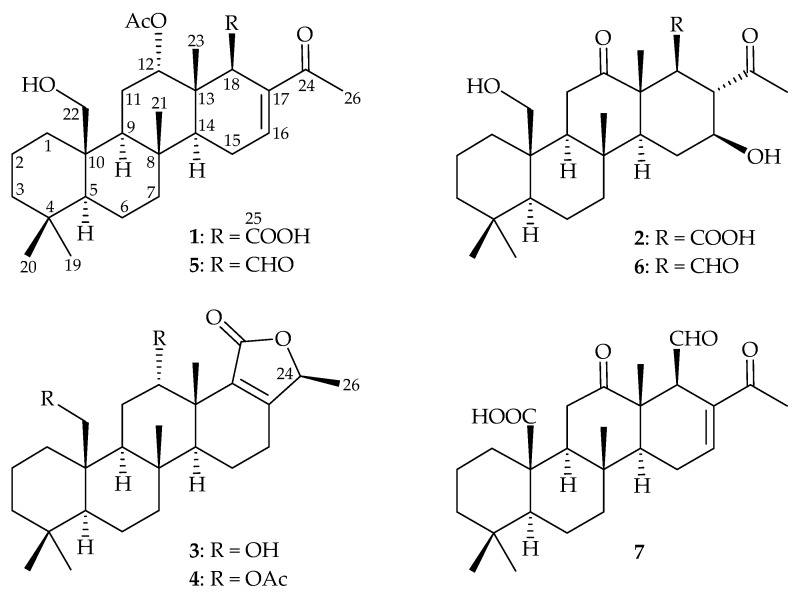
The structures of lendenfeldaranes A–D (**1**–**4**), 12α-acetoxy-22-hydroxy-24-methyl-24-oxo- scalar-16-en-25-al (**5**), felixin F (**6**), and 24-methyl-12,24,25-trioxoscalar-16-en-22-oic acid (**7**).

Since the first scalarane-type derivative, scalarin, was originally isolated from *Cocaspongia scalaris* [1], more than three hundred scalarane sesterterpenoids have been obtained from cyanobacteria and marine organisms [2,3]. Compounds of this type demonstrate a wide spectrum of interesting biological properties, such as anti-inflammation [4], cytotoxicity [5,6,7], anti-feedant [8,9,10], anti-microbial activity [11,12], ichthyotoxicity [13], anti-tubercular activity [14], anti-HIV [15], and inhibition of the nuclear hormone receptor [16]. In order to seek novel anti-proliferative substances from marine organisms, a chemical and bioactive investigation was carried out on the organic extracts of a marine sponge identified as *Lendenfeldia* sp. (family—Thorectidae). The ethyl acetate (EtOAc) extract of *Lendenfeldia* sp. was found to exhibit anti-proliferative activity against human cancer cell lines, including human acute lymphoblastic leukemia (MOLT-4), human chronic myelogenous leukemia (K-562), human histiocytic lymphoma (U-937), and human T-cell lymphoblastic lymphoma (SUP-T1) with IC_50_ values < 0.625 μg/mL. The bioassay-guided isolation, combined with an NMR spectroscopic pattern-based procedure, was used to explore the anti- proliferative scalarane substances, and led to the isolation four new 24-homoscalaranes, lendenfeldaranes A–D (**1**–**4**), along with three known metabolites, 12α-acetoxy-22-hydroxy-24- methyl-24-oxoscalar-16-en-25-al (**5**) [17], felixin F (**6**) [18], and 24-methyl-12,24,25-trioxoscalar-16- en-22-oic acid (**7**) [17]. In the current study, the comprehensive workflow of isolation, structure elucidation and an anti-proliferative evaluation were implemented on scalaranes **1**–**7** (Figure 1).

## 2. Results and Discussion

Lendenfeldarane A (**1**) was obtained as an amorphous powder and assigned the molecular formula C_28_H_42_O_6_ (eight degrees of unsaturation) from its (+)-HRESIMS at *m*/*z* 497.28736 [M + Na]^+^ (calcd. for C_28_H_42_O_6_ + Na, 497.28712). The ^1^H NMR data of **1** (Table 1), showed six singlet methyls at δ_H_ 0.75, 0.86, 0.96, 1.16, 2.14, and 2.29, one olefinic proton at δ_H_ 6.90 (1H, br s), and one oxymethine proton at δ_H_ 4.77 (1H, br s). The diastereotopic geminal protons at δ_H_ 3.85 (1H, d, *J* = 11.6 Hz) and 4.02 (1H, d, *J =* 11.6 Hz) were assumed to be an oxymethylene group. Analyses of the ^13^C and distortionless enhancement by polarization transfer (DEPT) spectra of **1** (Table 1) revealed the existence of 28 carbon resonances, including six methyls, eight sp^3^ methylenes (including one oxymethylene), five sp^3^ methines (including one oxymethine), four sp^3^ quaternary carbons, one sp^2^ methine, and four sp^2^ quaternary carbons (including three carbonyls). Based on the ^1^H and ^13^C NMR spectra, **1** was found to possess an acetoxy group (δ_H_ 2.14, 3H, s; δ_C_ 170.4, C; 21.5, CH_3_). An additional unsaturated functionality was indicated by ^13^C resonances at δ_C_ 139.7 (CH-16) and 137.9 (C-17), suggesting the presence of a trisubstituted olefin. Thus, four degrees of unsaturation were accounted for, and the above NMR data—as well as the unassigned degrees of unsaturation of **1**—implied a tetracyclic analogue.

**Figure 2:**
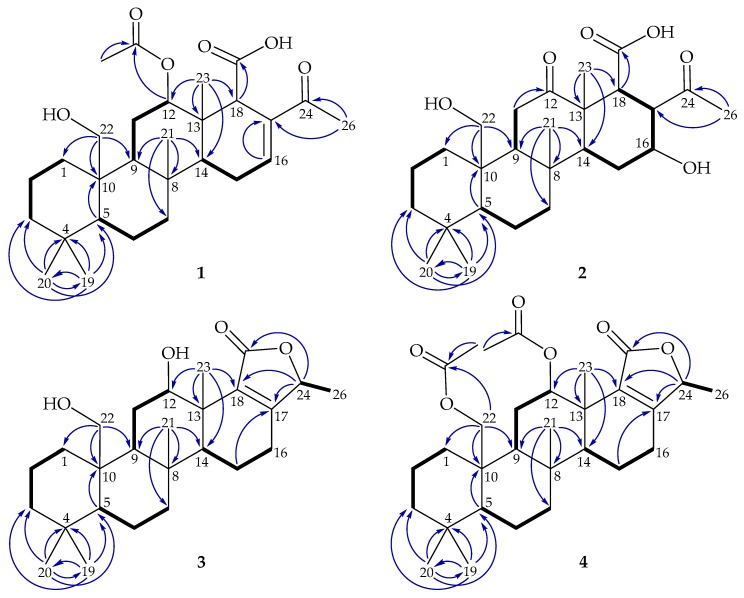
The key COSY correlations (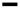) and heteronuclear multiple bond correlation (HMBC) (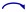) of **1**–**4**.

The gross structure of **1** was further established from its 2D NMR spectra. From the coupling information in the COSY spectrum of **1** (Figure 2), it was possible to establish four partial structure units between H_2_-1/H_2_-2/H_2_-3, H-5/H_2_-6/H_2_-7, H-9/H_2_-11/H-12, and H-14/H_2_-15/H_2_-16. The heteronuclear multiple bond correlation (HMBC) spectrum connected these substructures by the connectivity between H-5/C-10; H-16/C-17; H_3_-20/C-3, C-4, C-5, C-19; H_3_-21/C-7, C-8, C-9, C-14; H_2_-22/C-1, C-9, C-10; H_3_-23/C-12, C-13, C-14, C-18; and H_3_-26/C-17, C-24, indicating a scalarane-type sesterterpenoid structure (Figure 2). Furthermore, the acetoxy and carboxylic acid groups positioned at C-12 and C-18 were determined by the HMBC, from H-12 to the acetate carbonyl at δ_C_ 170.4 and from H-18 to C-25 (δ_C_ 175.1), respectively.

**Figure 3:**
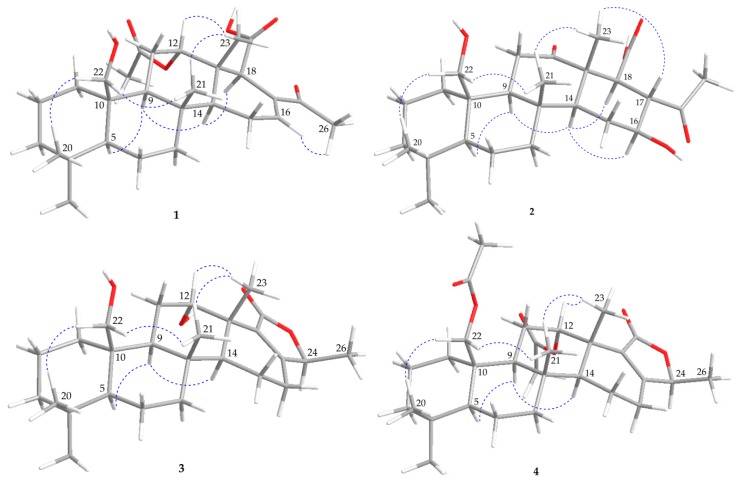
The selected NOESY correlations (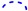) of **1**–**4**.

The relative stereochemistry of **1** was elucidated by correlations in the NOESY experiment. Using the conventional method for analyzing the stereochemistry, the α*-* and β*-*configurations were assigned at H-5 and C-10-hydroxymethyl, respectively, to anchor the stereochemical analysis. In the NOESY spectrum of **1** (Figure 3), H-9 correlated with H-5, but not with H_3_-21 and H_2_-22, suggesting that these two protons (H-5 and H-9) were situated on the same face and were α-oriented, and that the Me-21 and C-10-hydroxymethyl groups were β-oriented at C-8 and C-10, respectively. H-14 exhibited correlations with H-9 and H-18, but not with H_3_-21 and H_3_-23, demonstrating that H-14 and H-18 were α-oriented. Additionally, the proton signal of a methyl group at δ_H_ 0.96 (H_3_-23) displayed a correlation with H-12 (δ_H_ 4.77), which indicated the β-orientations of Me-23 and H-12. The NOESY spectrum showed a correlation between H_3_-26 and H-16, revealing the *E* geometry of the C-16/17 carbon–carbon double bond. It was found that the NMR data of **1** were similar to those of a known scalarane analogue, 12*α*-acetoxy-22-hydroxy-24-methyl-24-oxoscalar-16-en-25-al (**5**), from an Australian sponge, *Lendenfeldia* sp. [17], except that the aldehyde group in **5** was replaced by a carboxylic acid group in **1**. Based on the above findings, the structure of **1** was accordingly assigned, as shown in Figure 1, and named lendenfeldarane A (Supplementary Materials, Figures S1–S8).

Compound **2** (lendenfeldarane B) was obtained as an amorphous powder and its molecular formula was determined as C_26_H_40_O_6_, based on a sodiated adduct ion peak [M + Na]^+^ at *m/z* 471.27171 in (+)-HRESIMS (calcd. for C_26_H_40_O_6_ + Na, 471.27142). The ^1^H NMR data of **2** (Table 1) showed five singlet methyls at δ_H_ 0.76, 0.87, 1.30, 1.34, and 2.40 and one oxymethine proton at δ_H_ 3.53 (1H, ddd, *J* = 10.8, 10.8, 4.8 Hz). The diastereotopic geminal protons at δ_H_ 3.93 (1H, dd, *J* = 11.4, 1.2 Hz) and 4.08 (1H, d, *J* = 11.4 Hz) were assumed to be an oxygenated methylene group. The ^13^C and DEPT data of **2** suggested the presence of 26 carbons that were similar to those of a known scalarane, felixin F (**6**) [18], including a carboxylic carbon at δ_C_ 172.4, two ketone carbons at δ_C_ 212.6 and 221.9, an oxymethine carbon at δ_C_ 72.7, and an oxymethylene carbon at δ_C_ 62.7. Analysis of these NMR data suggested that compounds **2** and **6** are closely related, with the only difference being that the β- aldehyde group at C-18 in **6** was replaced by a β-carboxylic acid group in **2**. Based on the analyses of the COSY, HMBC, and NOESY spectra, as well as the specific rotation data (**2**: [α]${}_{D}^{20}$ +49 (*c* 0.99, CHCl_3_), **6**: [α]${}_{D}^{20}$ +55 (*c* 0.04, CHCl_3_); ref [18] **6**: [α]${}_{D}^{25}$ +54 (*c* 0.4, CHCl_3_)), Compound **2** was finally assigned, as shown in Figure 1, and named as lendenfeldarane B (Supplementary Materials, Figures S9–S16).

The molecular formula of lendenfeldarane C (**3**) was determined as C_26_H_40_O_4_ from an [M + Na]^+^ ion at m/z 439.28188 (calcd. for C_26_H_40_O_4_ + Na, 439.28174) and NMR data (Table 2), indicating seven degrees of unsaturation. The ^1^H NMR data of **3** showed four singlet methyls at δ_H_ 0.78, 0.86, 1.08, 1.13, one doublet methyl at δ_H_ 1.37 (J = 6.5 Hz), and two oxymethine protons at δ_H_ 4.60 (1H, br s) and 4.79 (1H, q, J = 6.5 Hz). The diastereotopic geminal protons at δ_H_ 3.92 (1H, d, J = 11.5 Hz) and 4.05 (1H, d, J = 11.5 Hz) were assumed to be an oxymethylene group. Analyses of the ^13^C NMR and DEPT spectrum of **3** revealed the existence of 26 carbon resonances, including five methyls, nine sp^3^ methylenes (including one oxymethylene), five sp^3^ methines (including two oxymethines), four sp^3^ quaternary carbons, and three sp^2^ quaternary carbons (including one ester carbonyl). Based on the ^13^C spectrum, **3** was found to possess an ester carbonyl (δ_C_ 172.6) and an unsaturated degree was indicated by the ^13^C chemical shifts at δ_C_ 133.5 (C-18) and 165.2 (C-17), suggesting the presence of a tetrasubstituted olefin. Thus, the above NMR data and the remaining five unsaturated degrees of **3** required a pentacyclic analogue.

**Figure 4:**
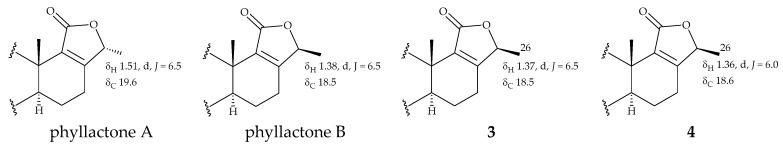
The ^1^H and ^13^C NMR chemical shifts in the methyl group in the α,β-unsaturated-γ-lactone moiety in phyllactones A and B and lendenfeldaranes C (**3**) and D (**4**).

The gross structure of **3** was constructed from its 2D NMR spectra. From the COSY spectrum (Figure 2), five partial structure units between H_2_-1/H_2_-2/H_2_-3, H-5/H_2_-6/H_2_-7, H-9/H_2_-11/H-12, H-14/ H_2_-15/H_2_-16, and H-24/H_3_-26 were established. The HMBC spectrum connected these fractional structures by the key correlations between H-5/C-10; H_2_-15/C-17; H_3_-20/C-3, C-4, C-5, C-19; H_3_-21/ C-7, C-8, C-9, C-14; H_2_-22/C-1, C-9, C-10; and H_3_-23/C-12, C-13, C-14, C-18, indicating a scalarane skeleton. The COSY correlation between H_3_-26/H-24 and the HMBC from H-24 to C-17, C-18, and C-25 allowed the establishment of a 5-methyl-2(5H)-furanone. In the NOESY experiement of **3** (Figure 3), H_3_-23 correlated with H_3_-21 and H-12, indicating the β-orientation of Me-23 and H-12, respectively. The orientation of Me-26 was determined to be β-oriented, based on the comparison of the NMR chemical shifts of Me-26 (δ_H_ 1.37, 3H, d, J = 6.5 Hz; δ_C_ 18.5) in **3** with those of previous reported scalarane analogues, phyllactones A (δ_H_ 1.51, 3H, d, J = 6.5 Hz; δ_C_ 19.6) and B (δ_H_ 1.38, 3H, d, J = 6.5 Hz; δ_C_ 18.5) (Figure 4) [5]. Hence, the structure of **3** was determined to be a new sesterterpenoid and this metabolite was found to be the 12-epi-compound of a known 24- homoscalarane, 23-hydroxy-20-methylscalarolide [19,20], and should be named lendenfeldarane C (Supplementary Materials, Figures S17–S24).

Compound **4** (lendenfeldarane D) has a molecular formula of C_30_H_44_O_6_, as established by (+)- HRESIMS at m/z 523.30301 (calcd. for C_30_H_44_O_6_ + Na, 523.30307). The ^1^H and ^13^C NMR data indicated that **4** possessed a structural skeleton similar to that of **3** (Table 2). Comparison of the ^1^H and ^13^C NMR spectra of **4** with those of **3** revealed that the C-12 oxymethine resonance at δ_C_ 69.9 observed in **3** was moved to δ_C_ 73.8 in **4**, and the C-22 oxymethene resonance at δ_C_ 63.0 observed in **3** was moved to δ_C_ 64.7 in **4**. Similarly, the ^1^H NMR spectrum of **4** displayed two additional acetate methyl signals at δ_H_ 1.97 and 2.07 (both 3H × s), relative to **3**. Therefore, the differences between compounds **3** and **4** are that the hydroxy groups at C-12 and C-22 in **3** were replaced by acetoxy groups in **4**. The gross structure of **4** is supported by the HMBC and COSY correlations (Figure 2). The stereochemical configuration was identical to that of other scalarane sesterterpenes based on the NOESY cross- peaks at H-5/H-9, H-9/H-14, H_3_-20/H-22, H-22/H_3_-21, H_3_-21/H_3_-23, and H_3_-23/H-12 (Figure 3). Thus, the structure of **4** was determined and named as lendenfeldarane D (Supplementary Materials, Figures S25–S32).

Based on the cytotoxicity that was demonstrated by the EtOAc extract of Lendenfeldia sp., all of the isolates **1**–**7** were assessed for their cytotoxicity toward the cancer cell lines MOLT-4, K-562, U-937, and SUP-T1 (Table 3). Compound **5** showed the most potent cytotoxicity toward MOLT-4 cells (IC_50_ = 0.31 μM). Since both compounds **5** and **7** were the major components, it was suggested that the cytotoxicity of the extract from Lendenfeldia sp. was attributed to these two scalaranes and the aldehyde groups in **5** and **7** played a significant role in their cytotoxicity.

## 3. Material and Methods

### 3.1. General Experimental Procedures

The optical rotation values were measured using a Jasco P-1010 digital polarimeter (Jasco, Tokyo, Japan). The IR spectra were obtained with a Thermo Scientific Nicolet iS5 FT-IR spectrophotometer (Thermo Fisher Scientific, Waltham, MA, USA). The NMR spectra were recorded on a 600 or a 400 MHz Jeol ECZ NMR (Jeol, Tokyo, Japan) and a 500 MHz Varian Unity INOVA NMR spectrometer (Varian, Palo Alto, CA, USA), using the residual CHCl_3_ signals (δ_H_ 7.26 ppm) and CDCl_3_ (δ_C_ 77.0 ppm) as the internal standards for ^1^H and ^13^C NMR, respectively. The coupling constants (J) are presented in Hz. ESIMS and HRESIMS were recorded using a Bruker 7 Tesla solariX FTMS system (Bruker, Bremen, Germany). The column chromatography was carried out with silica gel (230–400 mesh; Merck, Darmstadt, Germany). The TLC was performed on plates that were precoated with Kieselgel 60 F_254_ (0.25 mm thick, Merck, Darmstadt, Germany), then sprayed with 10% H_2_SO_4_ solution, followed by heating to visualize the spots. The normal-phase HPLC (NP-HPLC) was performed using a system comprising a pump (L-7110; Hitachi, Tokyo, Japan), an injection port (Rheodyne, 7725; Rohnert Park, CA, USA), and a semi-preparative normal-phase column (YMC- Pack SIL, 250 × 20 mm, 5 μm; Sigma-Aldrich, St. Louis, MO, USA). The reverse-phase HPLC (RP- HPLC) was performed using a system comprising a pump (L-2130; Hitachi), a photodiode array detector (L-2455; Hitachi), an injection port (Rheodyne; 7725), and a reverse-phase column (Luna 5 μm, C18(2) 100Å AXIA Packed, 250 × 21.2 mm; Phenomenex, Torrance, CA, USA).

### 3.2. Animal Material

The specimens of the marine sponge *Lendenfeldia* sp. were collected by hand, using self-contained underwater breathing apparatus (SCUBA), while diving off the coast of Southern Taiwan on 5 September 2012, and stored in a freezer until extraction. The sponge material was identified by Dr. Yusheng M. Huang, Department of Marine Recreation, National Penghu University of Science and Technology, Taiwan, by comparison—as described in a previous publication [21]. A voucher specimen (NMMBA-TWSP-12006) was deposited in the National Museum of Marine Biology and Aquarium, Pingtung, Taiwan.

### 3.3. Extraction and Isolation

The sliced bodies of *Lendenfeldia* sp. (wet weight 1.21 kg) were extracted with EtOAc. The EtOAc layer (5.09 g) was separated on silica gel and eluted using a mixture of *n*-hexane and EtOAc (stepwise, 100:1–pure EtOAc) to yield 11 fractions A–K. Fraction F was separated by NP-HPLC, using a mixture of *n*-hexane and EtOAc (3:1, flow rate: 3.0 mL/min) to afford seven fractions F1–F7. Fraction F3 was separated by RP-HPLC using a mixture of MeOH and H_2_O (7:3, flow rate: 5 mL/min) to afford **4** (1.2 mg). Fraction G was chromatographed on silica gel and eluted using *n*-hexane/ acetone (8:1—pure acetone) to afford eight fractions G1–G8. Fraction G3 was separated by NP-HPLC using a mixture of *n*-hexane and acetone (2.5:1, flow rate: 3.0 mL/min) to afford 10 fractions G3A– G3J, including Compound **7** (fraction G3C, 57.5 mg). Fraction G3D was separated by RP-HPLC using a mixture of MeOH and H_2_O (8:2, flow rate: 5 mL/min) to afford **3** (0.8 mg). Fraction H was separated on silica gel and eluted using a mixture of *n*-hexane and acetone (6:1–2:1) to obtain 14 fractions H1–H14. Fraction H5 was re-purified by NP-HPLC, using a mixture of *n*-hexane and acetone (5:1, flow rate: 3.0 mL/min) to afford **5** (49.2 mg) and **6** (2.0 mg), respectively. Fraction K was separated by NP-HPLC using a mixture of *n*-hexane and acetone (2:1, flow rate: 3.0 mL/min) to afford **1** (11.4 mg) and **2** (7.6 mg).

Lendenfeldarane A (**1**): amorphous powder; [α]${}_{D}^{20}$ +17 (*c* 0.99, CHCl_3_); IR (ATR) ν_max_ 3552–2420 (broad), 3467, 1716, 1667 cm^−1^; ^1^H (CDCl_3_, 400 MHz) and ^13^C (CDCl_3_, 100 MHz) NMR data, see Table 1; ESIMS: *m/z* 497 [M + Na]^+^; HRESIMS: *m/z* 497.28736 (calcd. for C_28_H_42_O_6_ + Na, 497.28712).

Lendenfeldarane B (**2**): amorphous powder; [α]${}_{D}^{20}$ +49 (*c* 0.99, CHCl_3_); IR (ATR) ν_max_ 3444–2309 (broad), 3399, 1740, 1730 cm^−1^; ^1^H (CDCl_3_, 600 MHz) and ^13^C (CDCl_3_, 150 MHz) NMR data, see Table 1; ESIMS: *m/z* 471 [M + Na]^+^; HRESIMS: *m/z* 471.27171 (calcd. for C_26_H_40_O_6_ + Na, 471.27142).

Lendenfeldarane C (**3**): amorphous powder; [α]${}_{D}^{20}$ +56 (*c* 0.04, CHCl_3_); IR (ATR) ν_max_ 3436, 1731 cm^−1^; ^1^H (CDCl_3_, 500 MHz) and ^13^C (CDCl_3_, 125 MHz) NMR data, see Table 2; ESIMS: *m/z* 439 [M + Na]^+^; HRESIMS: *m/z* 439.28188 (calcd. for C_26_H_40_O_4_ + Na, 439.28174).

Lendenfeldarane D (**4**): amorphous powder; [α]${}_{D}^{20}$ +38 (*c* 0.05, CHCl_3_); IR (ATR) ν_max_ 1738, 1672 cm^−1^; ^1^H (CDCl_3_, 600 MHz) and ^13^C (CDCl_3_, 150 MHz) NMR data, see Table 2; ESIMS: *m/z* 523 [M + Na]^+^; HRESIMS: *m/z* 523.30301 (calcd. for C_30_H_44_O_6_ + Na, 523.30307).

12α-Acetoxy-22-hydroxy-24-methyl-24-oxoscalar-16-en-25-al (**5**): amorphous powder; [α]${}_{D}^{20}$ +24 (*c* 2.46, CHCl_3_); IR (ATR) ν_max_ 1732, 1702, 1662 cm^−1^; ^1^H (CDCl_3_, 400 MHz) and ^13^C (CDCl_3_, 100 MHz) NMR data were found to be in complete agreement with previous report [17]; ESIMS: *m/z* 481 [M + Na]^+^.

Felixin F (**6**): amorphous powder; [α]${}_{D}^{20}$ +55 (*c* 0.04, CHCl_3_) (ref. [18] [α]${}_{D}^{25}$ +54 (*c* 0.4, CHCl_3_)); IR (ATR) ν_max_ 3430, 1701 cm^−^^1^; ^1^H (CDCl_3_, 400 MHz) and ^13^C (CDCl_3_, 100 MHz) NMR data were found to be in complete agreement with previous report [18]; ESIMS: *m/z* 455 [M + Na]^+^.

24-Methyl-12,24,25-trioxoscalar-16-en-22-oic acid (**7**): amorphous powder; [α]${}_{D}^{20}$ +68 (*c* 0.04, CHCl_3_) (ref. [17] [α]${}_{D}^{21}$ +33.5 (*c* 1, CHCl_3_)); IR (ATR) ν_max_ 3468–2388 (broad), 1732, 1702, 1662 cm^–^^1^; ^1^H (CDCl_3_, 400 MHz) and ^13^C (CDCl_3_, 100 MHz) NMR data were found to be in complete agreement with previous report [17]; ESIMS: *m/z* 451 [M + Na]^+^.

### 3.4. MTT Cell Proliferative Assay

The anti-proliferative properties of the metabolites against a limited panel of human tumor cell lines, including MOLT-4, K-562, U-937, and SUP-T1, were assayed. The cell lines were purchased from the American Type Culture Collection (ATCC). The cells were seeding at 2 × 10^4^ and were cultured in 96-well plates. The cytotoxic effect of the tested compounds was determined by the (3- (4,5-dimethylthiazol-2-yl)-2,5-diphenyltetrazolium bromide (MTT)-cell proliferation assay (Sigma- M2128; Sigma-Aldrich, St. Louis, MO, USA) after 72 h. A total of 50 μL of MTT solution was added to each well for 1 h and an ELISA reader (Anthoslabtec Instrument, Salzburg, Austria) was used (OD = OD_570_ − OD_620_) for the IC_50_ value, calculated with CalcuSyn software.

## 4. Conclusions

The marine sponge belonging to the genus *Lendenfeldia* has proven to be a prolific producer of bioactive metabolites, especially sesterterpenoids with a scalarane skeleton. In the present study, seven 24-homoscalaranes were obtained from the *Lendenfeldia* sp. that was collected from the waters of Southern Taiwan, including four new 24-homoscalaranes, lendenfeldaranes A–D (**1**–**4**), along with three known analogues, 12α-acetoxy-22-hydroxy-24-methyl-24-oxo-scalar-16-en-25-al (**5**), felixin F (**6**), and 24-methyl-12,24,25-trioxoscalar-16-en-22-oic acid (**7**). The anti-cancer assessments indicated that **5**–**7** showed the most promising anti-proliferative activities against tumor cells. The structure-activity relationship (SAR) discussions also suggested the pivotal role of 18-aldehyde functionality in the activity against leukemia and lymphoma. Overall, these results can support the potential use of the marine sponge, genus *Lendenfeldia*, as a therapeutic agent in the treatment of cancer. We have therefore begun to culture this potentially useful sponge in tanks, using our highly developed aquaculture technology, for the extraction of natural products in order to establish a stable supply of bioactive materials, which will also protect the natural population and habitats from over-exploitation.

## Figures and Tables

**Table 1:** The ^1^H and ^13^C NMR data for 24-homoscalaranes **1** and **2** (CDCl_3_).

C/H	1		2	
	δ_H_ (*J* in Hz) ^a^	δ_C_ Multiple ^b^	δ_H_ (*J* in Hz) ^c^	δ_C_ Multiple ^d^
1	2.12 m; 0.48 ddd (12.8, 12.8, 3.2)	34.4, CH_2_	2.13 m; 0.80 m	33.8, CH_2_
2	1.50 m	17.8, CH_2_	1.44 m; 1.65 m	17.9, CH_2_
3	1.19 m; 1.43 m	41.6, CH_2_	1.18 m; 1.43 m	41.4, CH_2_
4		33.0, C		33.0, C
5	0.94 m	56.8, CH	0.95 br d (12.6)	56.8, CH
6	1.43 m	18.3, CH_2_	1.53 m	18.2, CH_2_
7	1.03 m; 1.82 ddd (12.8, 3.2, 3.2)	42.2, CH_2_	0.96 m; 1.91 ddd (13.2, 3.6, 3.6)	42.1, CH_2_
8		37.8, C		38.7, C
9	1.50 m	49.3, CH	1.26 m	62.8, CH
10		41.7, C		42.8, C
11	2.07 m	24.9, CH_2_	2.62 dd (12.6, 1.8); 3.34 dd (14.4, 12.6)	39.0, CH_2_
12	4.77 br s	75.7, CH		221.9, C
13		38.9, C		52.8, C
14	1.31 m	52.2, CH	1.21 m	58.0, CH
15	2.27 m	23.3, CH_2_	1.65 m; 1.94 ddd (12.6, 4.2, 1.8)	30.1, CH_2_
16	6.90 br s	139.7, CH	3.53 ddd (10.8, 10.8, 4.8)	72.7, CH
17		137.9, C	3.22 dd (12.0, 10.8)	54.8, CH
18	3.79 br s	48.0, CH	3.18 d (12.0)	51.3, CH
19	0.75 s	21.9, CH_3_	0.76 s	21.7, CH_3_
20	0.86 s	33.8, CH_3_	0.87 s	33.7, CH_3_
21	1.16 s	16.1, CH_3_	1.30 s	16.4, CH_3_
22	3.85 d (11.6); 4.02 d (11.6)	62.9, CH_2_	3.93 dd (11.4, 1.2); 4.08 d (11.4)	62.7, CH_2_
23	0.96 s	15.5, CH_3_	1.34 s	15.3, CH_3_
24		199.3, C		212.6, C
25		175.1, C		172.4, C
26	2.29 s	25.3, CH_3_	2.40 s	33.4, CH
OAc-12		170.4, C		
	2.14 s	21.5, CH_3_		

^a^ 400 MHz. ^b^ 100 MHz. ^c^ 600 MHz. ^d^ 150 MHz.

**Table 2:** The ^1^H and ^13^C NMR data for 24-homoscalaranes **3** and **4** (CDCl_3_).

C/H	3		4	
	δ_H_ (*J* in Hz) ^a^	δ_C_ Multiple ^b^	δ_H_ (*J* in Hz) ^c^	δ_C_ Multiple ^d^
1	2.16 m; 0.80 m	34.0, CH_2_	2.01 m; 0.52 ddd (13.8, 13.8, 3.0)	34.7, CH_2_
2	1.63 m	18.4, CH_2_	1.63 m	18.2, CH_2_
3	1.18 m; 1.43 m	41.7, CH_2_	1.15 ddd (9.0, 9.0, 4.2); 1.43 m	41.5, CH_2_
4		33.0, C		33.0, C
5	1.05 m	56.8, CH	1.01 dd (13.8, 3.6)	57.1, CH
6	1.56 m; 1.91 m	16.9, CH_2_	1.56 m; 1.93 m	17.0, CH_2_
7	1.10 m; 1.89 m	42.0, CH_2_	1.11 ddd (12.0, 12.0, 4.2); 1.92 m	41.9, CH_2_
8		37.6, C		37.5, C
9	1.56 m	52.3, CH	1.26 m	53.2, CH
10		41.8, C		40.2, C
11	1.89 m; 2.18 m	27.1, CH_2_	1.99 m; 2.20 m	23.3, CH_2_
12	4.60 br s	69.9, CH	5.54 t (3.0)	73.8, CH
13		40.2, C		38.4, C
14	1.60 m	50.0, CH	1.55 m	51.2, CH
15	2.18 m; 2.35 m	24.1, CH_2_	2.23 m; 2.39 m	24.0, CH_2_
16	1.46 m; 1.56 m	18.0, CH_2_	1.60 m	18.0, CH_2_
17		165.2, C		163.6, C
18		133.5, C		132.6, C
19	0.78 s	21.8, CH_3_	0.83 s	21.9, CH_3_
20	0.86 s	33.9, CH_3_	0.89 s	33.7, CH_3_
21	1.08 s	16.3, CH_3_	0.98 s	16.4, CH_3_
22	3.92 d (11.5); 4.05 d (11.5)	63.0, CH_2_	4.15 dd (12.0, 1.2); 4.58 d (12.0)	64.7, CH_2_
23	1.13 s	21.7, CH_3_	1.19 s	21.3, CH_3_
24	4.79 q (6.5)	78.2, CH	4.78 q (6.0)	77.7, CH
25		172.6, C		171.1, C
26	1.37 d (6.5)	18.5, CH_3_	1.36 d (6.0)	18.6, CH_3_
OAc-12				169.9, C
			1.97 s	21.2, CH_3_
OAc-22				170.9, C
			2.07 s	21.2, CH_3_

^a^ 500 MHz. ^b^ 125 MHz. ^c^ 600 MHz. ^d^ 150 MHz.

**Table 3:** The anti-proliferative effects of scalaranes **1**–**7**.

Compound	Cell lines IC_50_ (μM)			
	MOLT-4	K-562	U-937	SUP-T1
1	39.54	NA	NA	33.02
2	34.93	NA	NA	NA
3	6.31	11.69	5.74	9.00
4	29.83	NA	NA	NA
5	0.31	3.04	2.35	5.90
6	5.67	9.71	6.97	12.33
7	1.49	1.04	5.88	7.49
Doxorubicin ^a^	0.02	0.13	0.04	0.09

^a^ Doxorubicin was used as a positive control; NA: not active at 20 μg/mL for 72 h.

## Supplementary Materials

The following are available online at https://www.mdpi.com/1660-3397/18/2/76/s1. HRESIMS, 1D, and 2D NMR spectra of compounds **1**–**4**.
