# Supplementary material for: Probing Anti-Proliferative 24-Homoscalaranes from a Sponge Lendenfeldia sp
Source: Mar Drugs. 2020 Jan 24;18(2):76. doi: 10.3390/md18020076 (PMC7074534; doi:10.3390/md18020076)
Supplement: Supplementary file 1 [file marinedrugs-18-00076-s001.pdf]

Supporting Information

No	Content	page
S1.	HRESIMS spectrum of compound **1**.	2
S2.	^{1}H NMR spectrum (400 MHz) of compound **1** in $CDCl_3$.	2
S3.	^{13}C NMR spectrum (100 MHz) of compound **1** in $CDCl_3$.	3
S4.	DEPT spectrum (100 MHz) of compound **1** in $CDCl_3$.	3
S5.	HSQC spectrum of compound **1** in $CDCl_3$.	4
S6.	HMBC spectrum of compound **1** in $CDCl_3$.	4
S7.	^{1}H–^{1}H COSY spectrum of compound **1** in $CDCl_3$.	5
S8.	NOESY spectrum of compound **1** in $CDCl_3$.	5
S9.	HRESIMS spectrum of compound **2**.	6
S10.	^{1}H NMR spectrum (600 MHz) of compound **2** in $CDCl_3$.	6
S11.	^{13}C NMR spectrum (150 MHz) of compound **2** in $CDCl_3$.	7
S12.	DEPT spectrum (150 MHz) of compound **2** in $CDCl_3$.	7
S13.	HSQC spectrum of compound **2** in $CDCl_3$.	8
S14.	HMBC spectrum of compound **2** in $CDCl_3$.	8
S15.	^{1}H–^{1}H COSY spectrum of compound **2** in $CDCl_3$.	9
S16.	NOESY spectrum of compound **2** in $CDCl_3$.	9
S17.	HRESIMS spectrum of compound **3**.	10
S18.	^{1}H NMR spectrum (500 MHz) of compound **3** in $CDCl_3$.	10
S19.	^{13}C NMR spectrum (125 MHz) of compound **3** in $CDCl_3$.	11
S20.	DEPT spectrum (125 MHz) of compound **3** in $CDCl_3$.	11
S21.	HSQC spectrum of compound **3** in $CDCl_3$.	12
S22.	HMBC spectrum of compound **3** in $CDCl_3$.	12
S23.	^{1}H–^{1}H COSY spectrum of compound **3** in $CDCl_3$.	13
S24.	NOESY spectrum of compound **3** in $CDCl_3$.	13
S25.	HRESIMS spectrum of compound **4**.	14
S26.	^{1}H NMR spectrum (600 MHz) of compound **4** in $CDCl_3$.	14
S27.	^{13}C NMR spectrum (150 MHz) of compound **4** in $CDCl_3$.	15
S28.	DEPT spectrum (150 MHz) of compound **4** in $CDCl_3$.	15
S29.	HSQC spectrum of compound **4** in $CDCl_3$.	16
S30.	HMBC spectrum of compound **4** in $CDCl_3$.	16
S31.	^{1}H–^{1}H COSY spectrum of compound **4** in $CDCl_3$.	17
S32.	NOESY spectrum of compound **4** in $CDCl_3$.	17

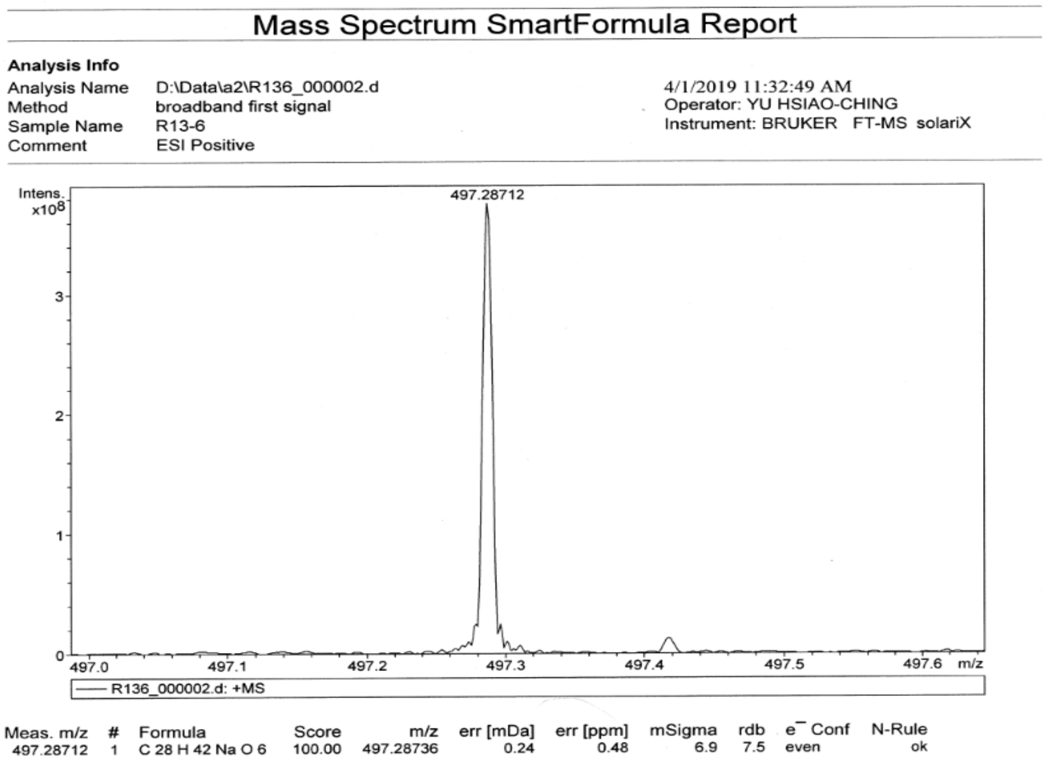

S1. HRESIMS spectrum of compound **1**.

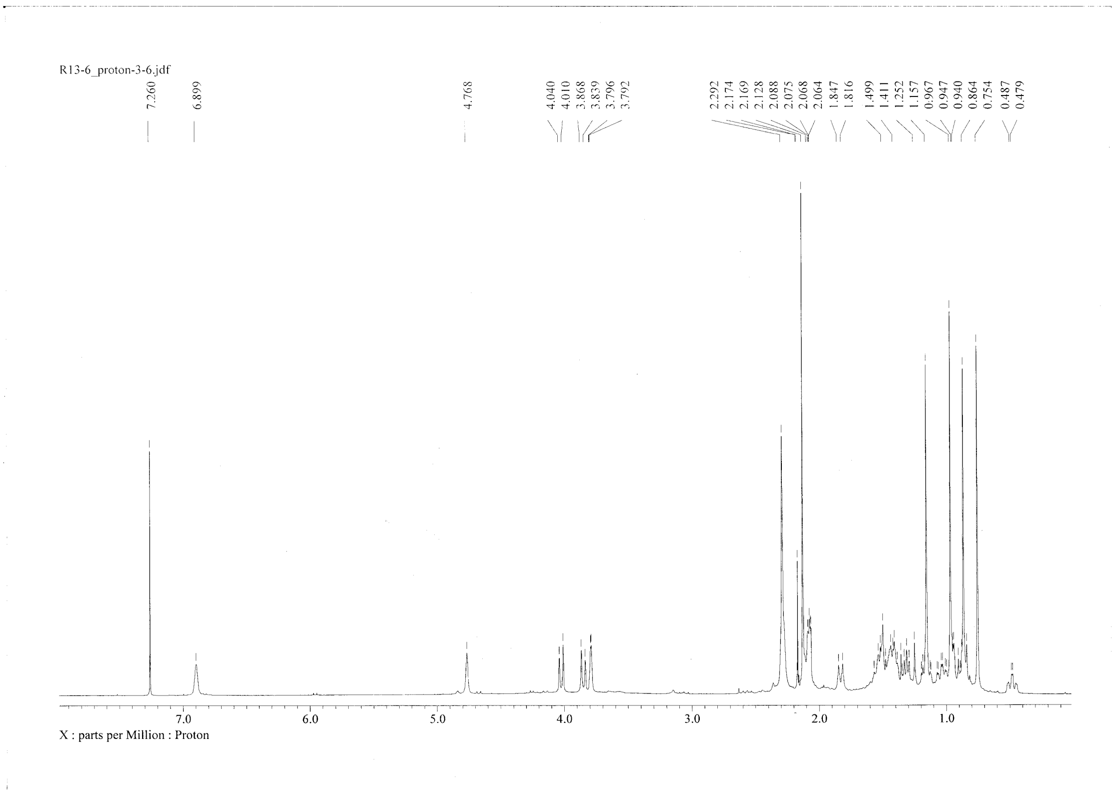

S2. ^{1}H NMR spectrum (400 MHz) of compound **1** in $CDCl_3$.

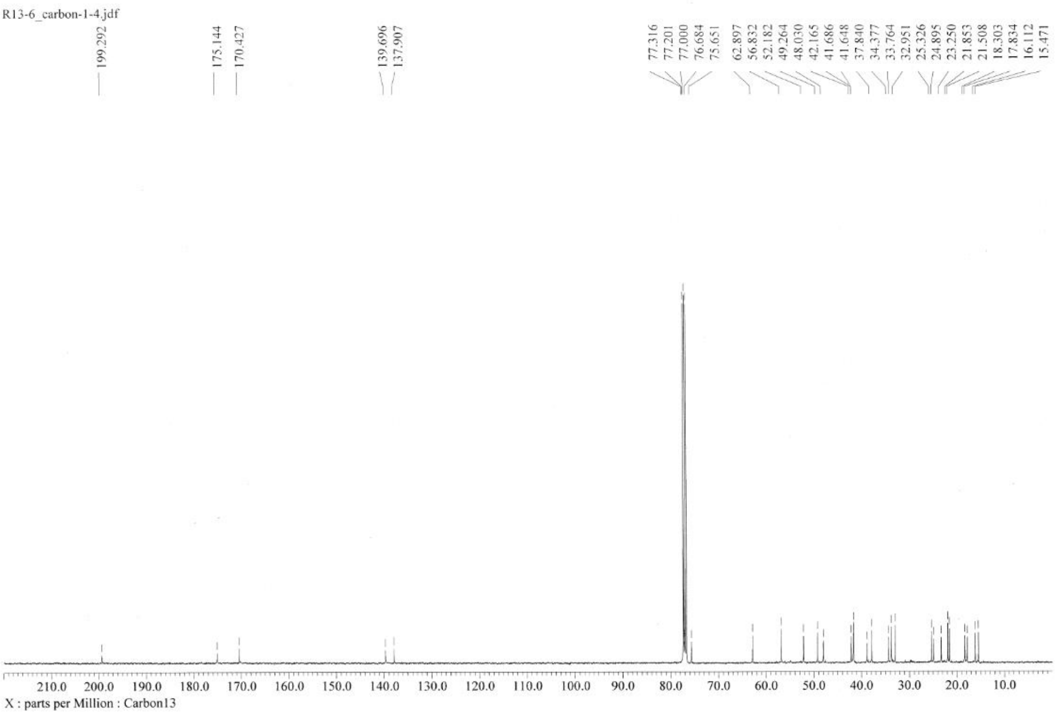

S3. ^{13}C NMR spectrum (100 MHz) of compound **1** in $CDCl_3$.

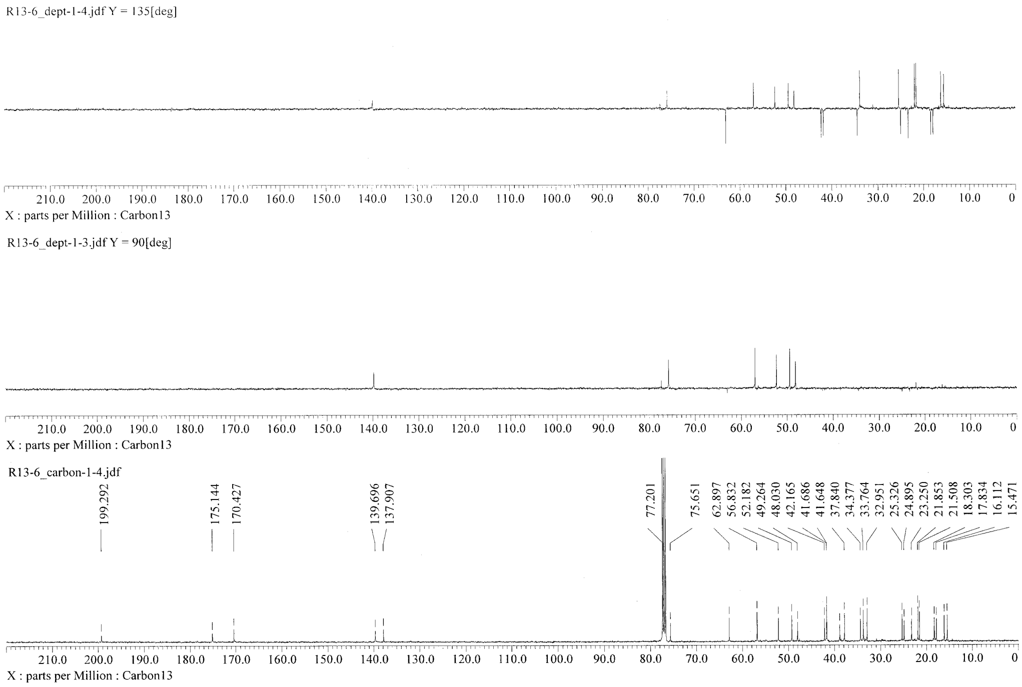

S4. DEPT spectrum (100 MHz) of compound **1** in CDC_3

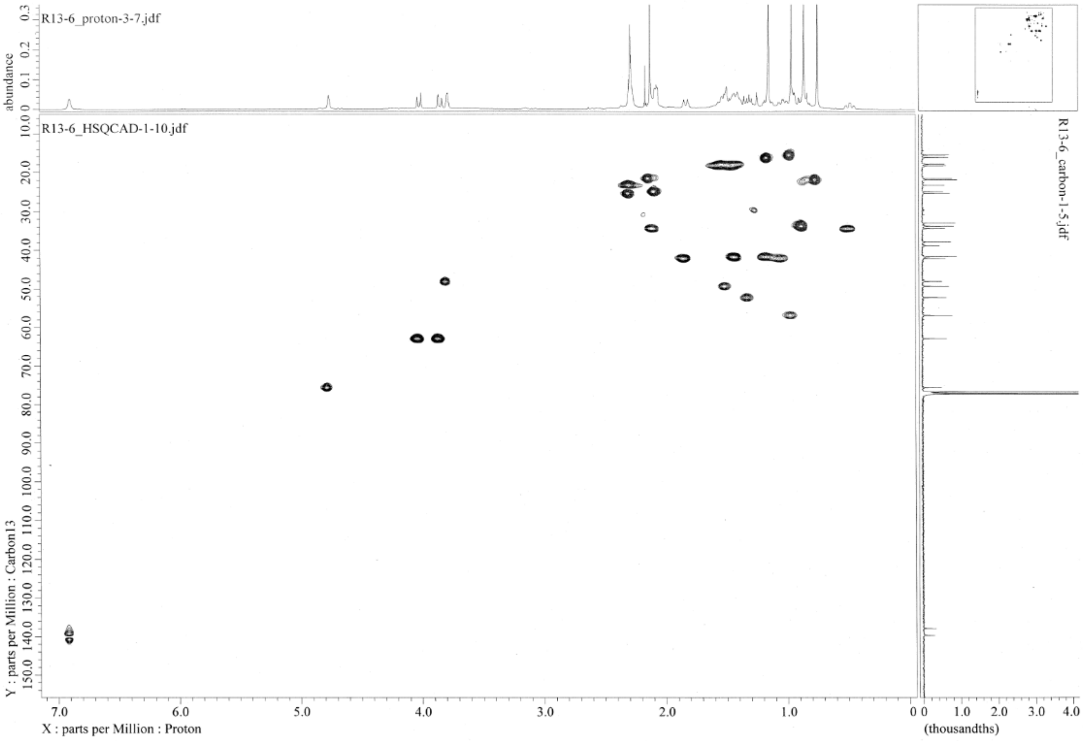

S5. HSQC spectrum of compound **1** in $CDCl_3$.

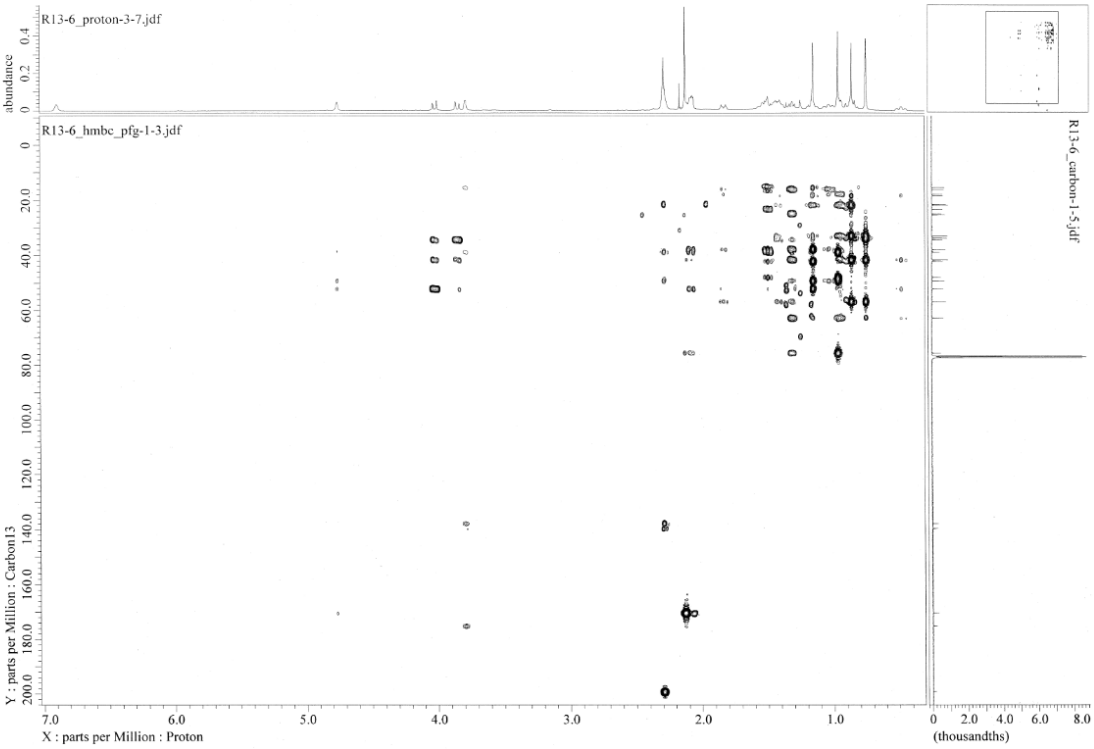

S6. HMBC spectrum of compound **1** in $CDCl_3$.

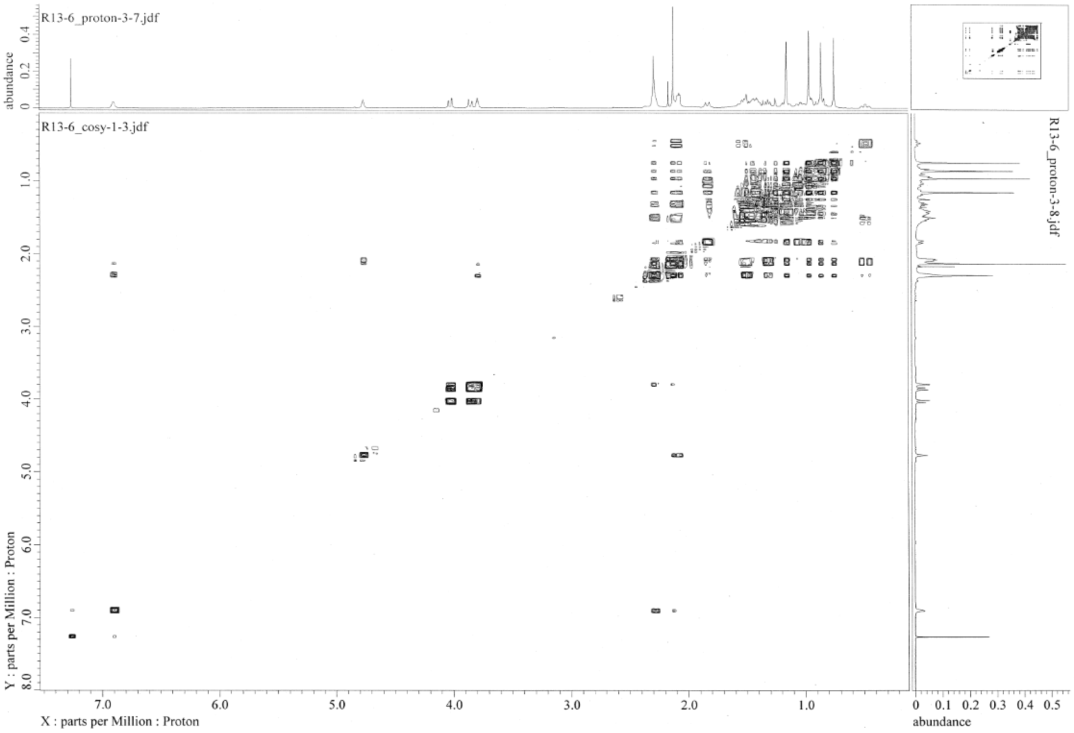

S7. ^{1}H-^{1}H COSY spectrum of compound **1** in $CDCl_3$.

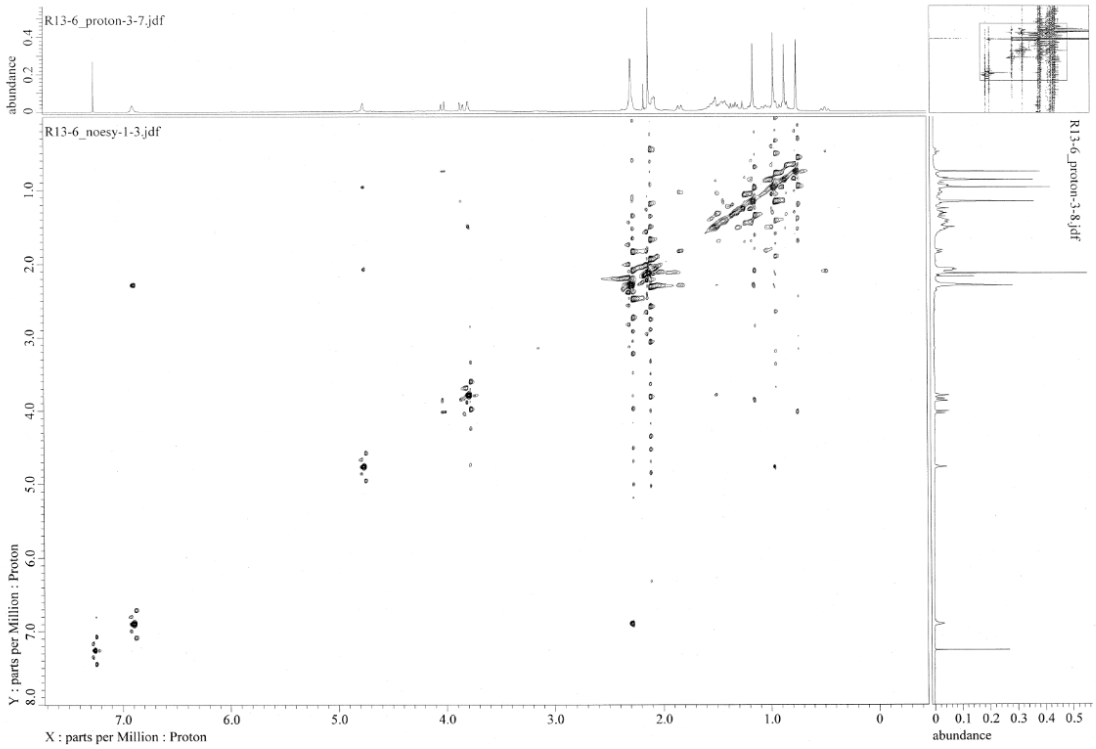

S8. NOESY spectrum of compound **1** in $CDCl_3$

Mass Spectrum SmartFormula Report

Analysis Info

Analysis Name	D:\Data\a2\R137_000004.d	4/2/2019 4:12:24 PM
Method	broadband first signal	Operator: YU HSIAO-CHING
Sample Name	R13-7	Instrument: BRUKER FT-MS solariX
Comment	ESI Positive	

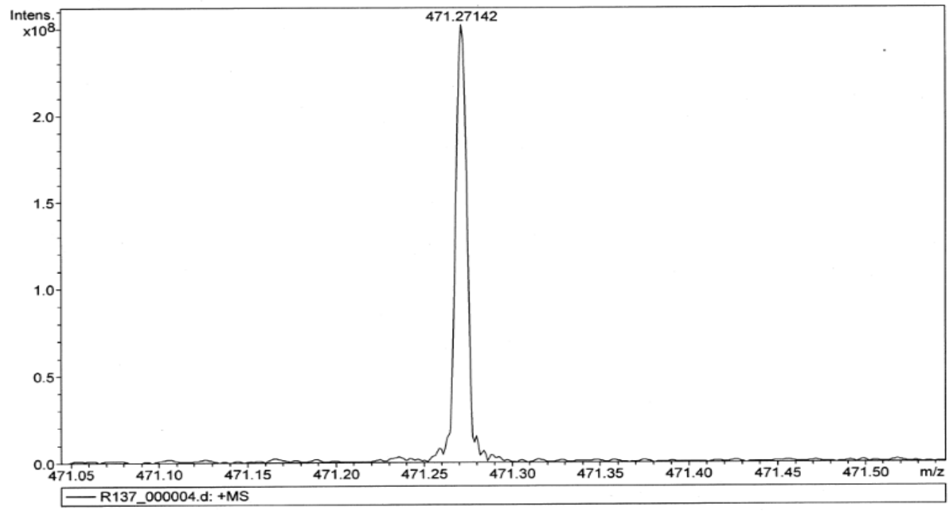

Meas. m/z	#	Formula	Score	m/z	err [mDa]	err [ppm]	mSigma	rdb	e⁻ Conf	N-Rule
471.27142	1	C 26 H 40 Na O 6	100.00	471.27171	0.29	0.61	5.0	6.5	even	ok

S9. HRESIMS spectrum of compound **2**.

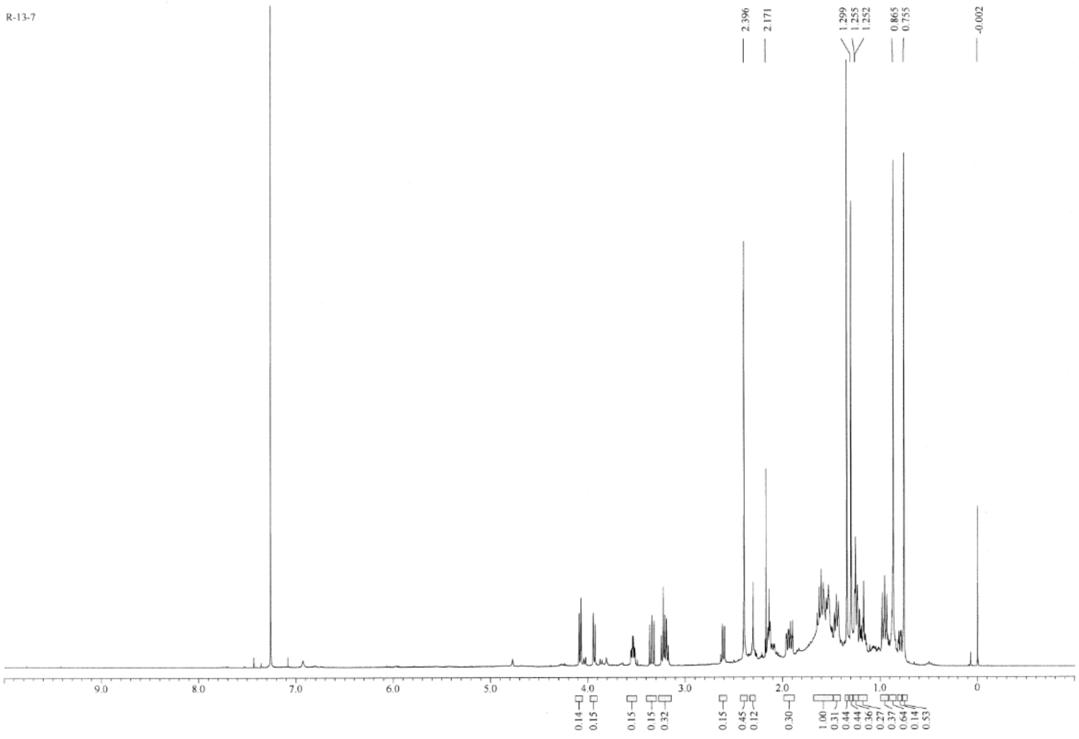

S10. ^{1}H NMR spectrum (600 MHz) of compound **2** in $CDCl_3$.

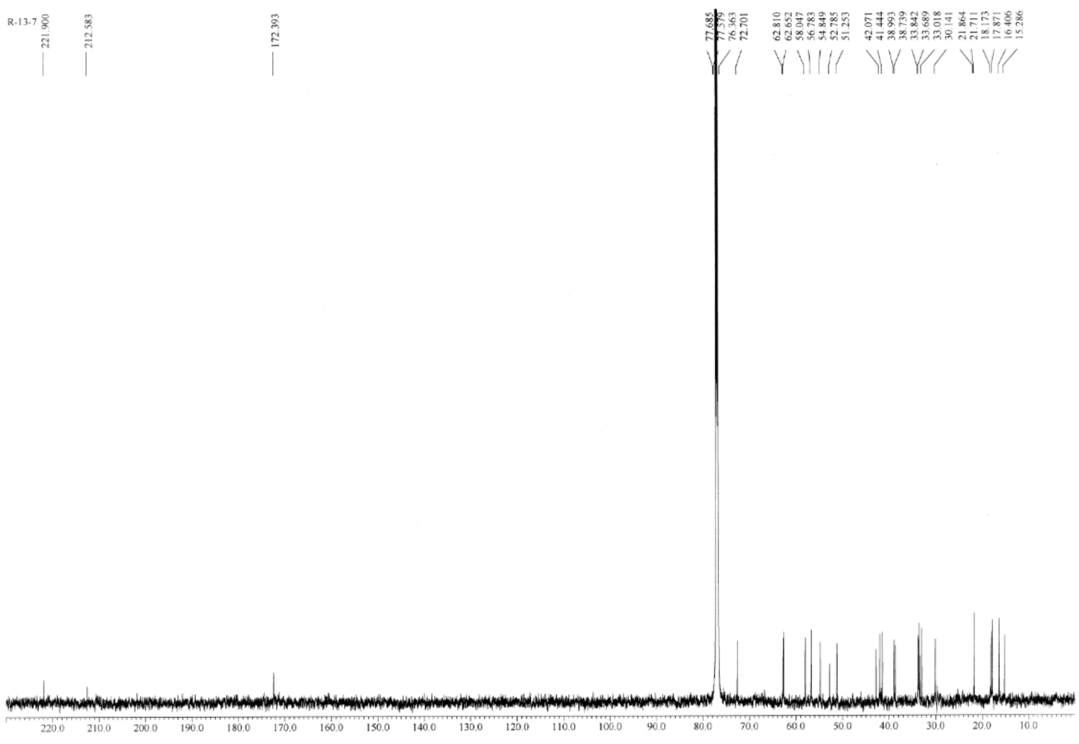

S11. ^{13}C NMR spectrum (150 MHz) of compound **2** in $CDCl_3$.

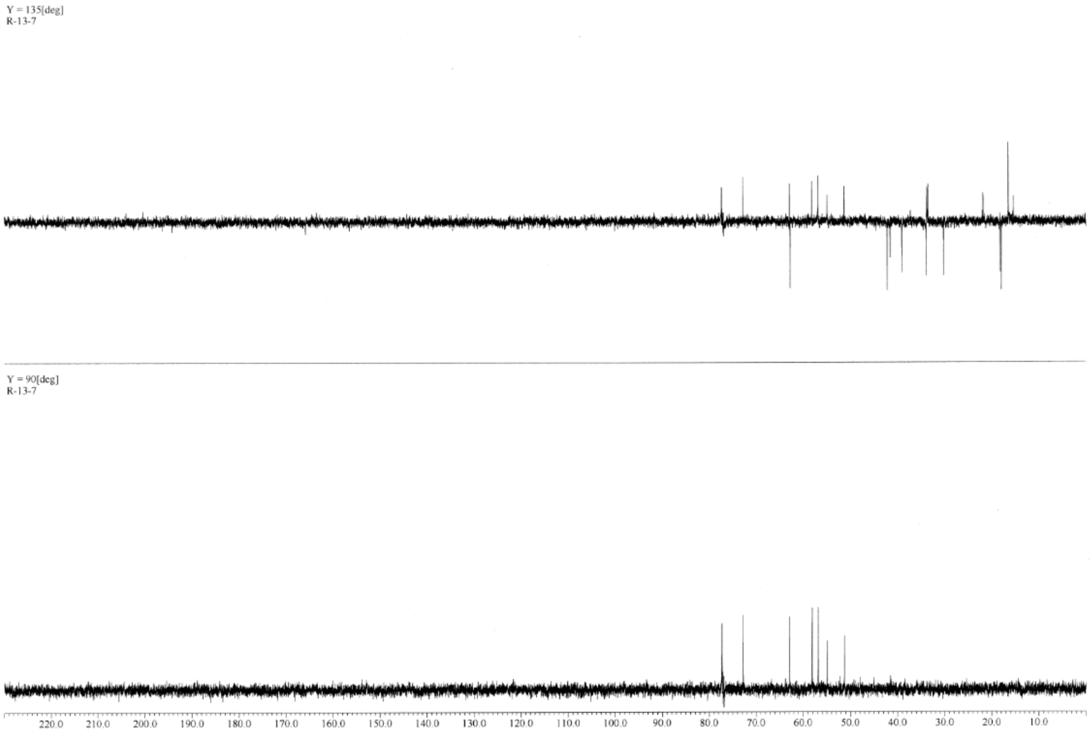

S12. DEPT spectrum (150 MHz) of compound **2** in $CDCl_3$.

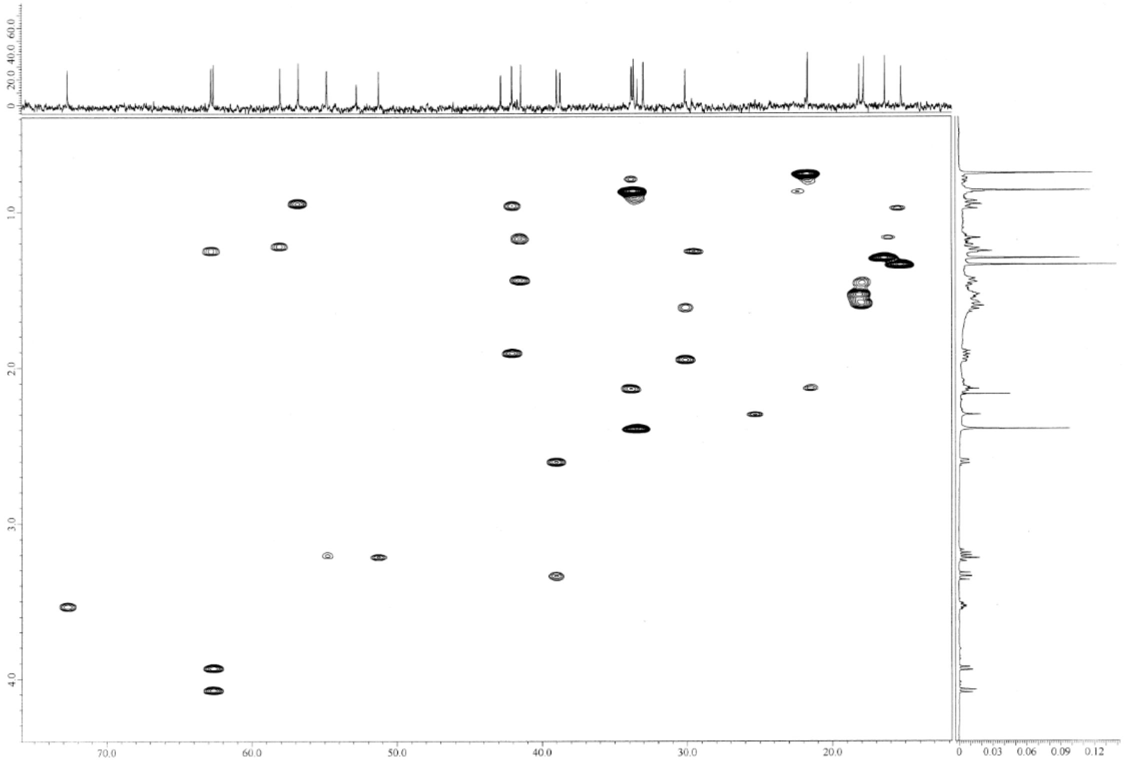

S13. HSQC spectrum of compound **2** in $CDCl_3$.

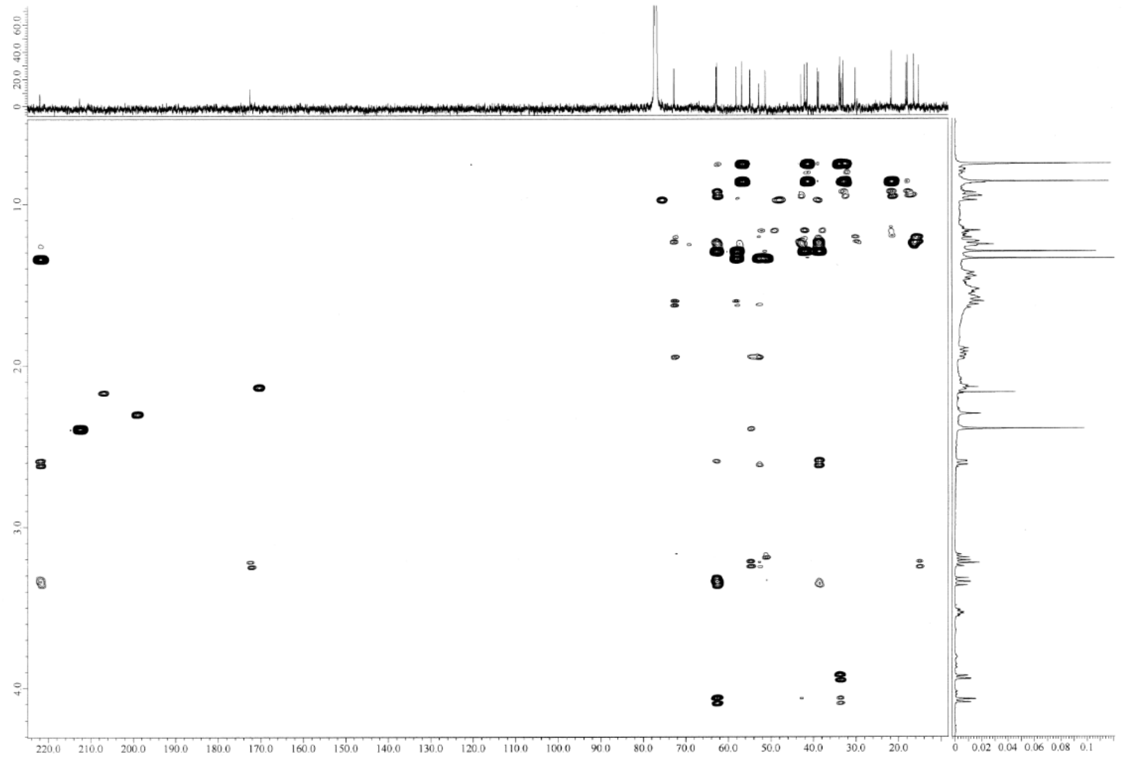

S14. HMBC spectrum of compound **2** in $CDCl_3$.

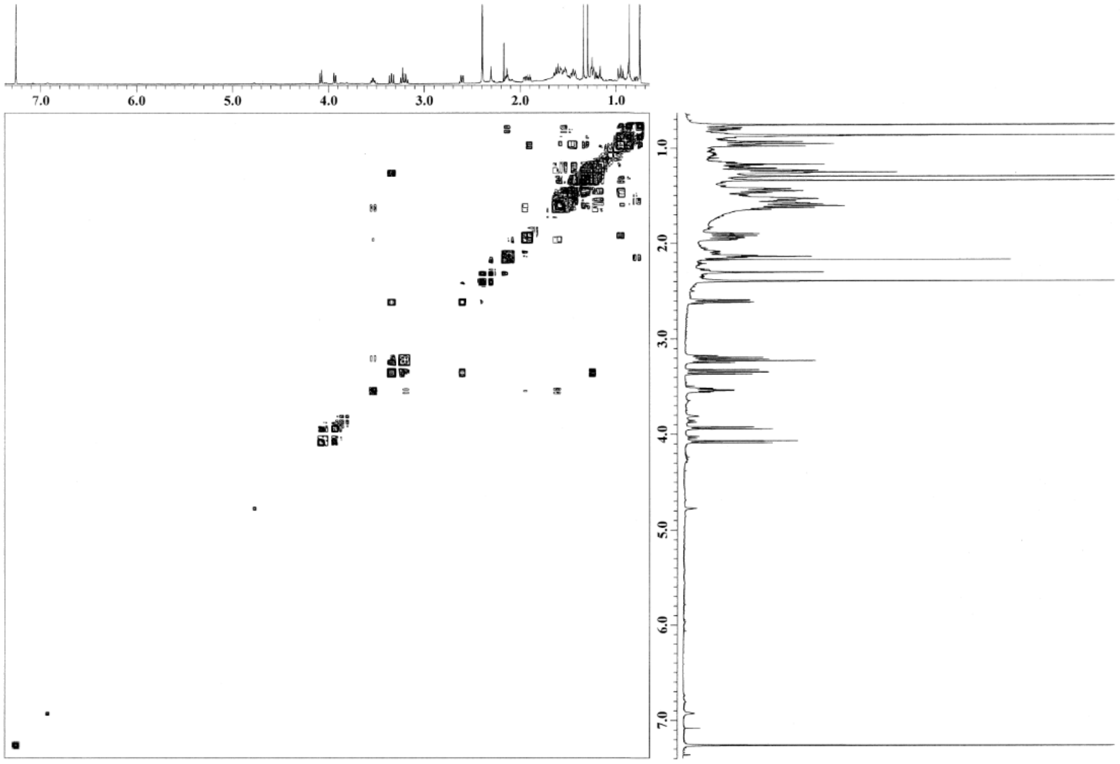

S15. ^{1}H-^{1}H COSY spectrum of compound **2** in $CDCl_3$.

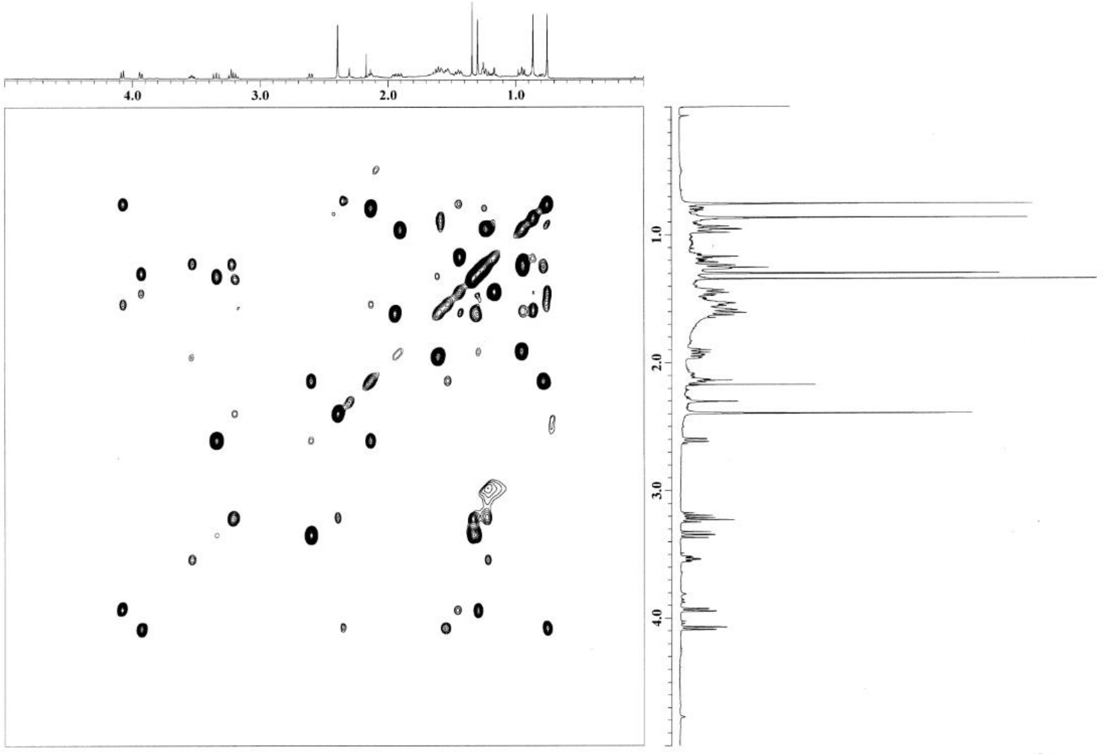

S16 NOESY spectrum of compound **2** in $CDCl_3$

Mass Spectrum SmartFormula Report

Analysis Info

Analysis Name	D:\Data\a2\R7348_000002.d	3/22/2019 3:36:10 PM
Method	broadband first signal	Operator: YU HSIAO-CHING
Sample Name	R7-3-4-8	Instrument: BRUKER FT-MS solariX
Comment	ESI Positive	

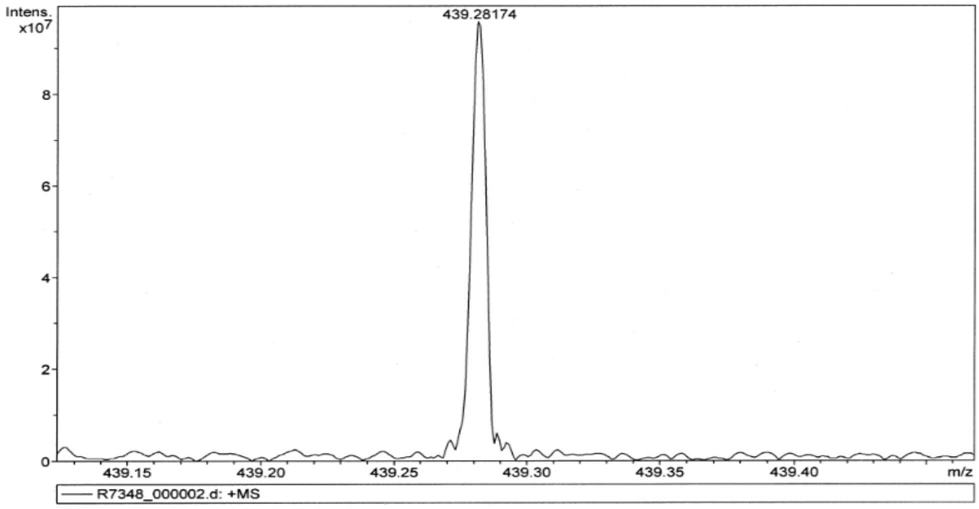

Meas. m/z	#	Formula	Score	m/z	err [mDa]	err [ppm]	mSigma	rdb	e⁻ Conf	N-Rule
439.28174	1	C 26 H 40 Na O 4	100.00	439.28188	0.15	0.33	10.7	6.5	even	ok

S17. HRESIMS spectrum of compound **3**.

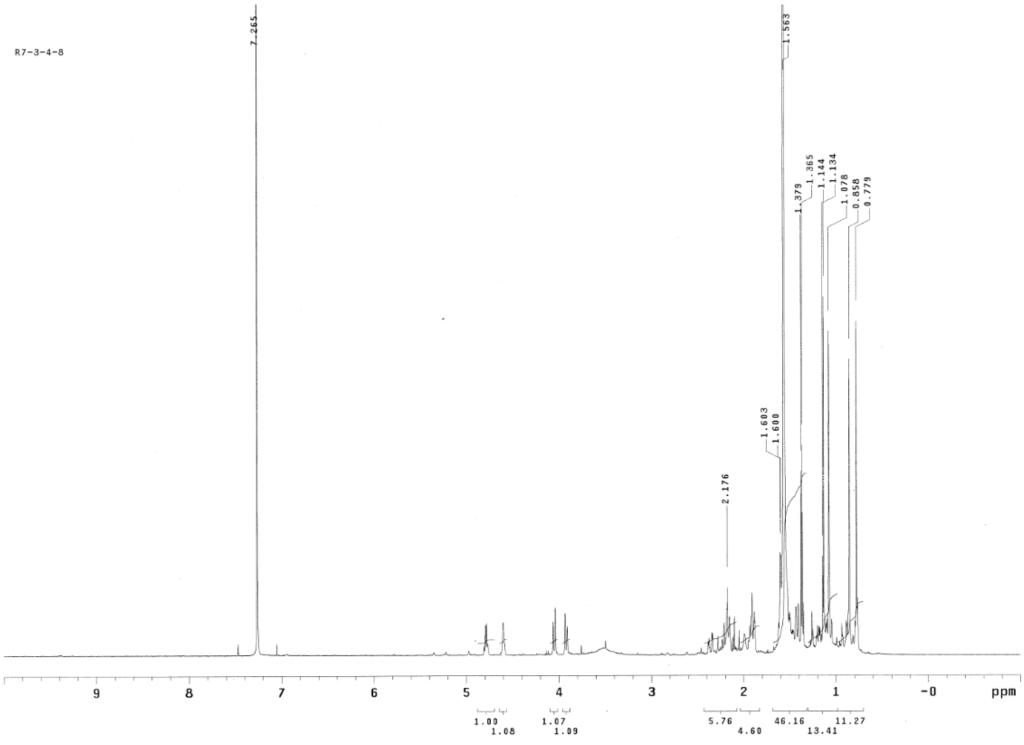

S18. ^{1}H NMR spectrum (500 MHz) of compound **3** in $CDCl_3$.

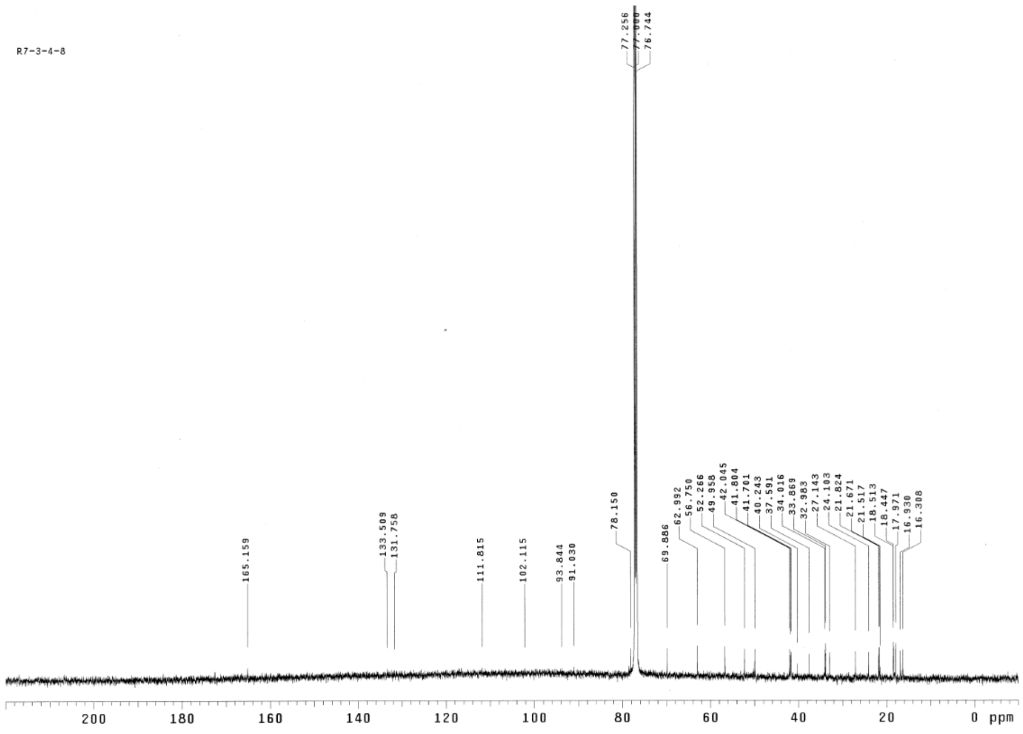

S19. ^{13}C NMR spectrum (125 MHz) of compound **3** in $CDCl_3$.

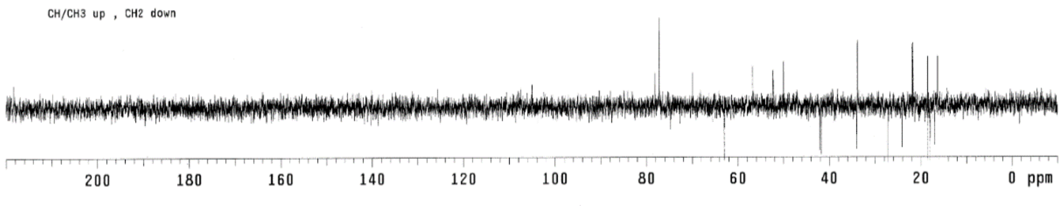

S20. DEPT spectrum (125 MHz) of compound **3** in $CDCl_3$.

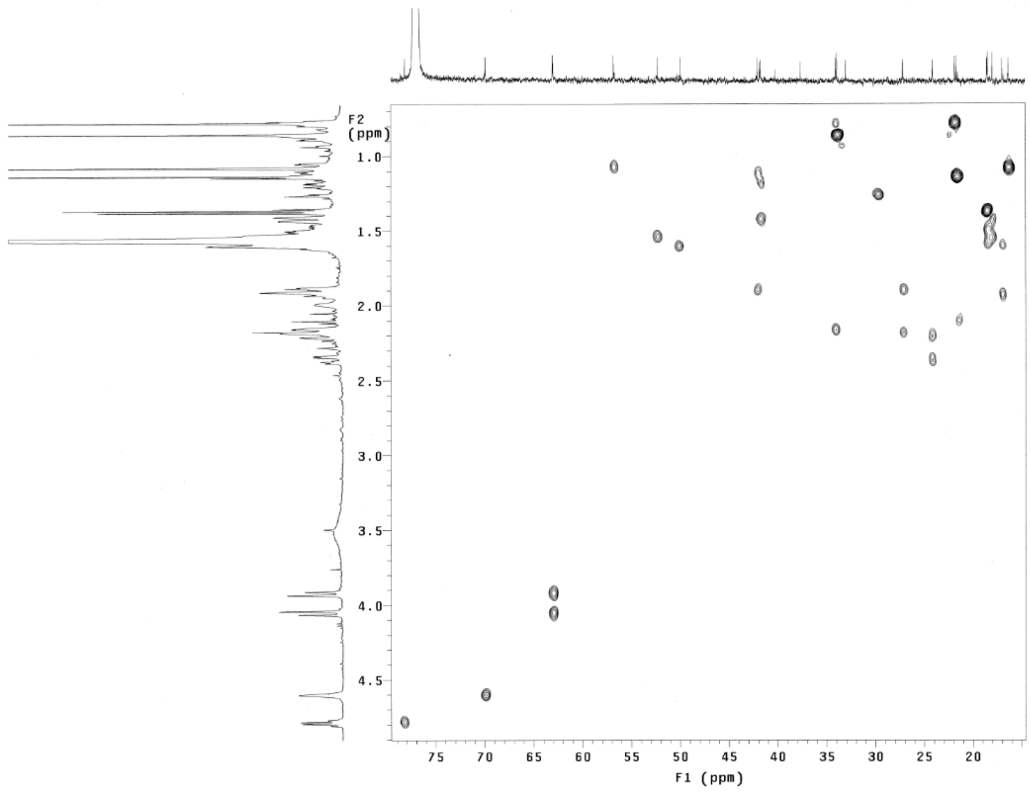

S21. HSQC spectrum of compound **3** in $CDCl_3$.

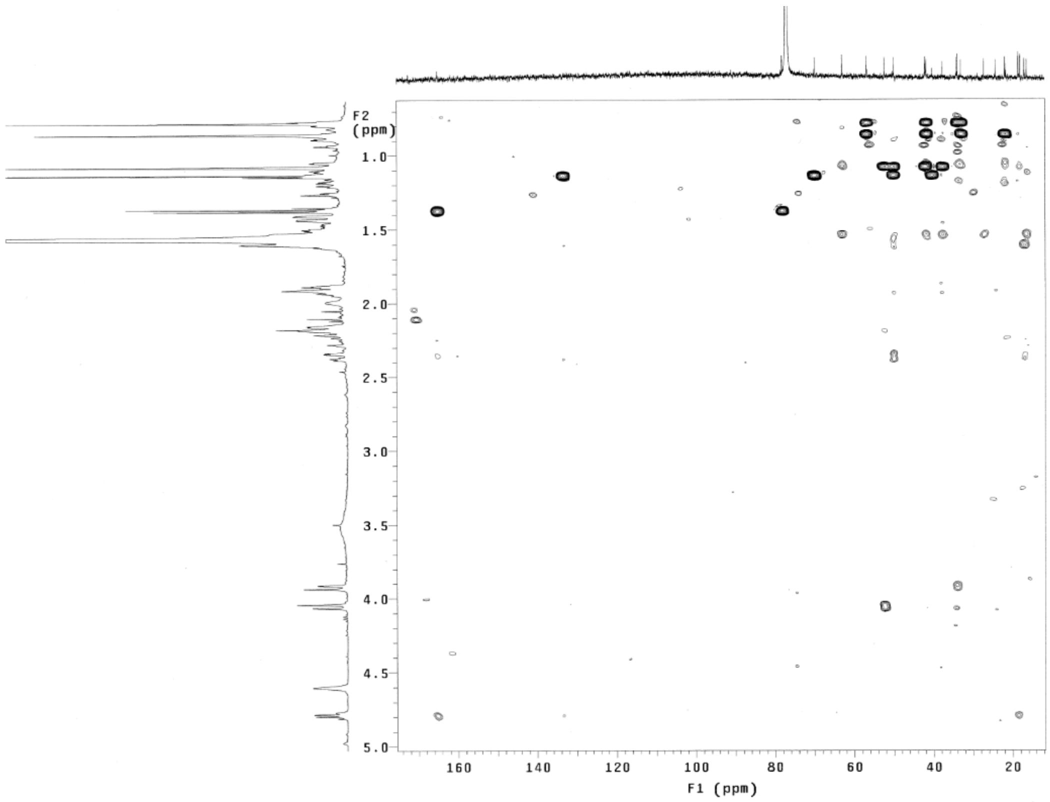

S22. HMBC spectrum of compound **3** in $CDCl_3$.

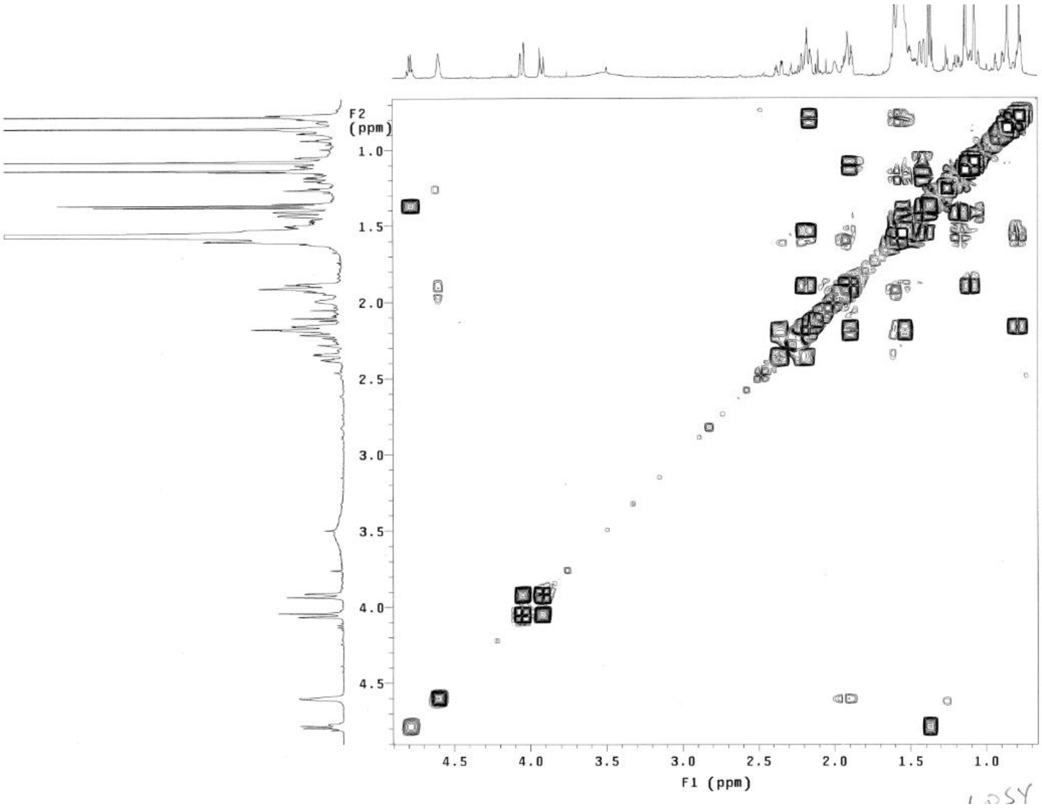

S23. ^{1}H-^{1}H COSY spectrum of compound **3** in $CDCl_3$.

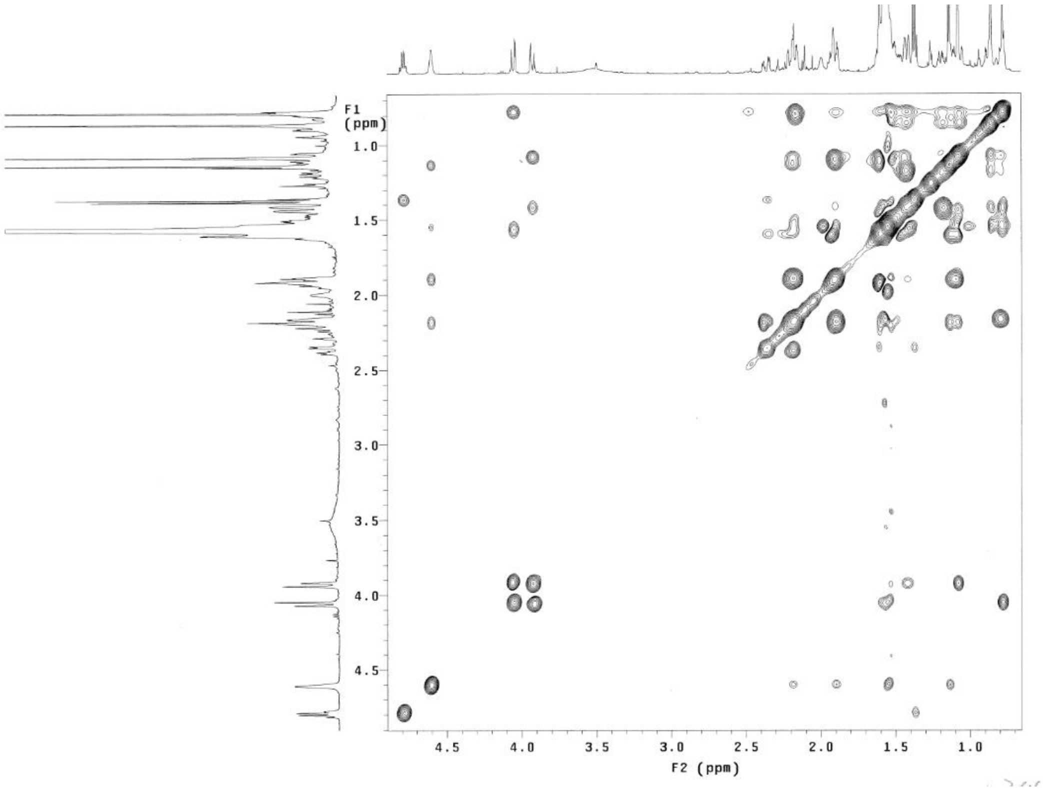

S24. NOESY spectrum of compound **3** in $CDCl_3$

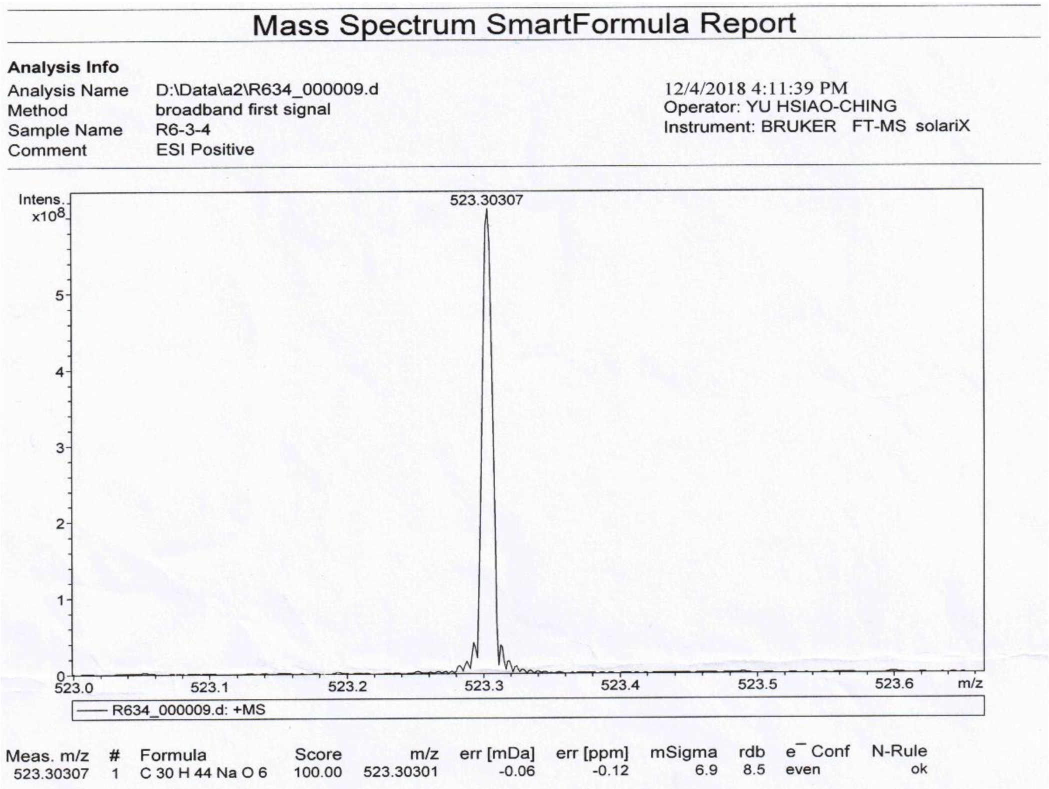

S25. HRESIMS spectrum of compound **4**.

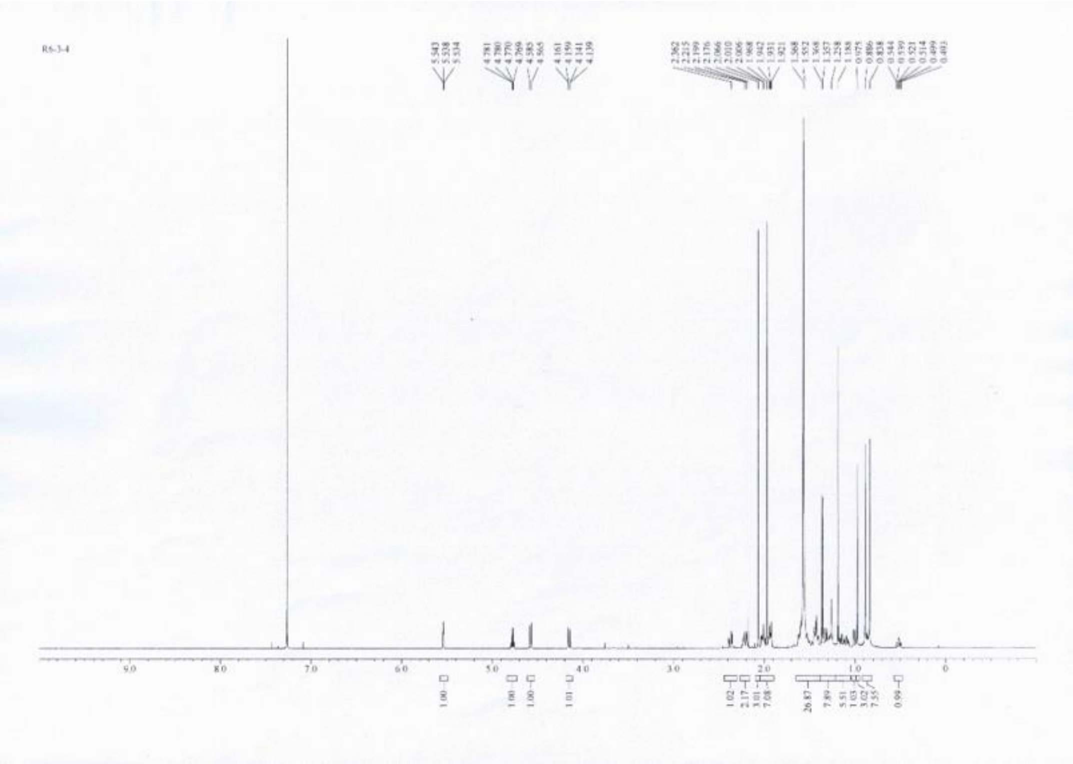

S26. ^{1}H NMR spectrum (600 MHz) of compound **4** in $CDCl_3$.

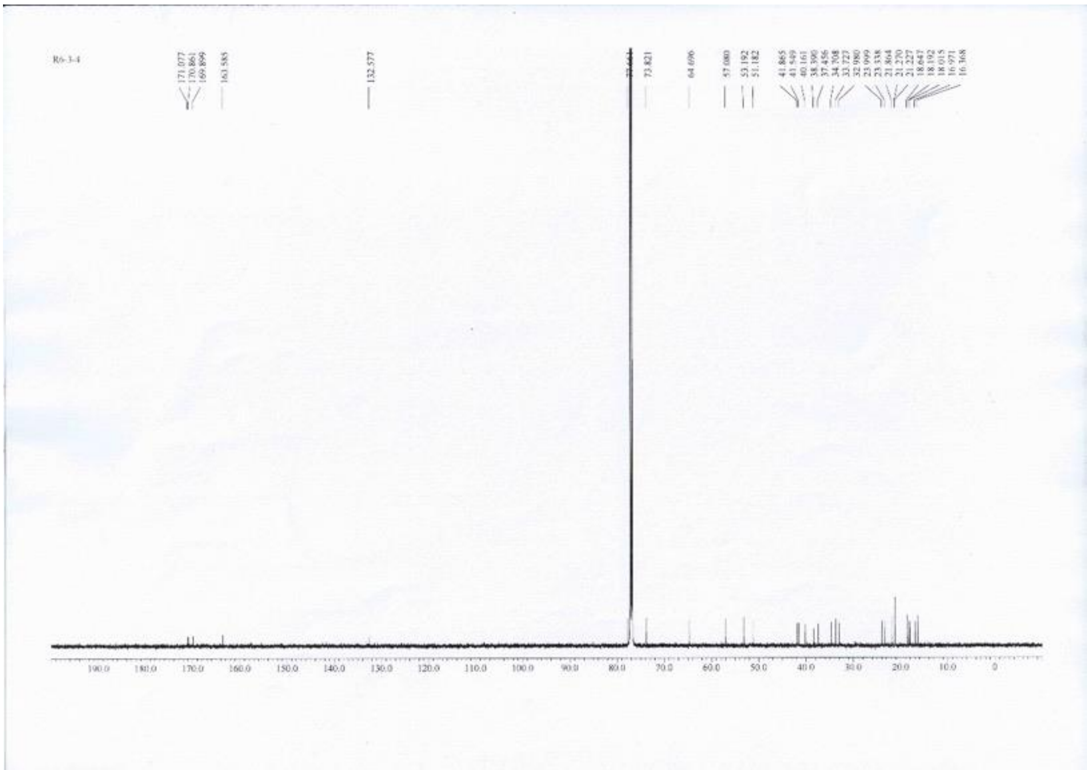

S27. ^{13}C NMR spectrum (150 MHz) of compound **4** in $CDCl_3$.

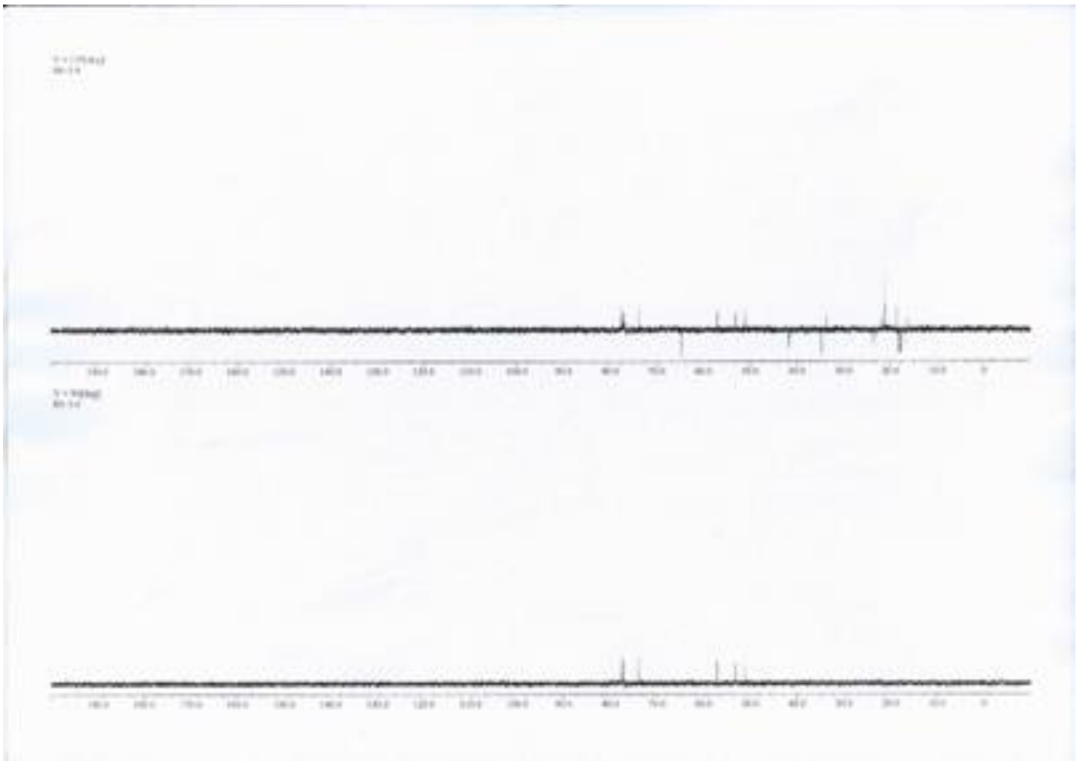

S28. DEPT spectrum (150 MHz) of compound **4** in $CDCl_3$.

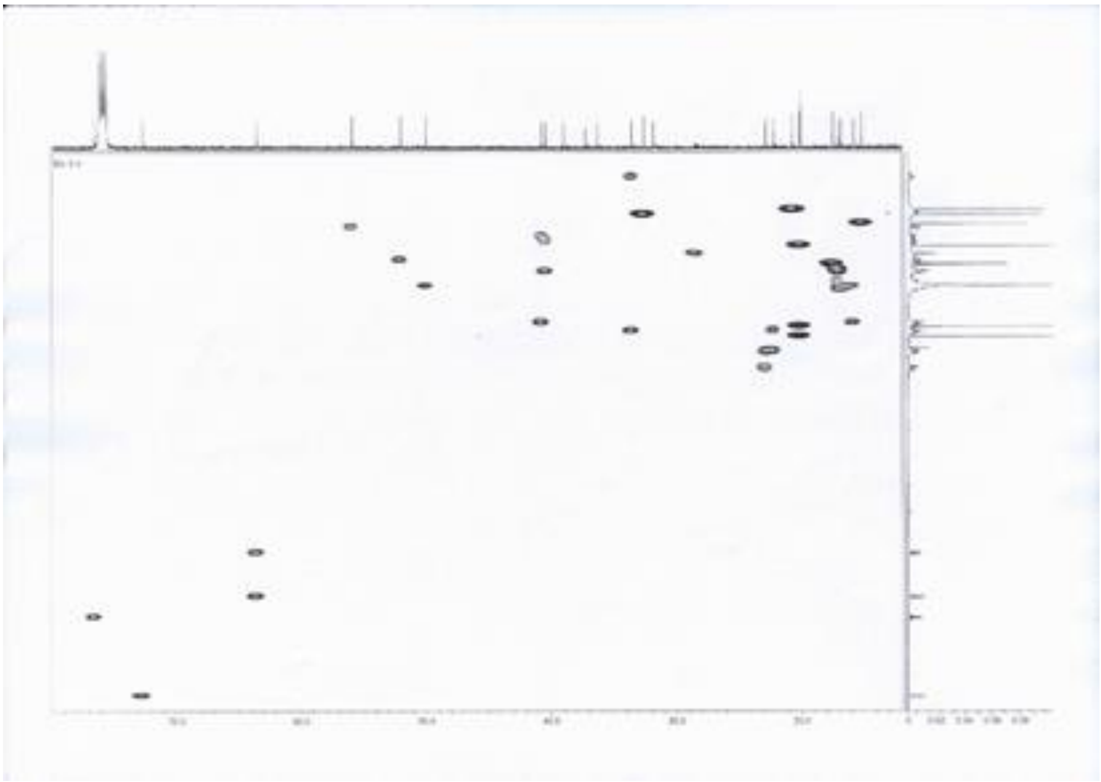

S29. HSQC spectrum of compound **4** in $CDCl_3$.

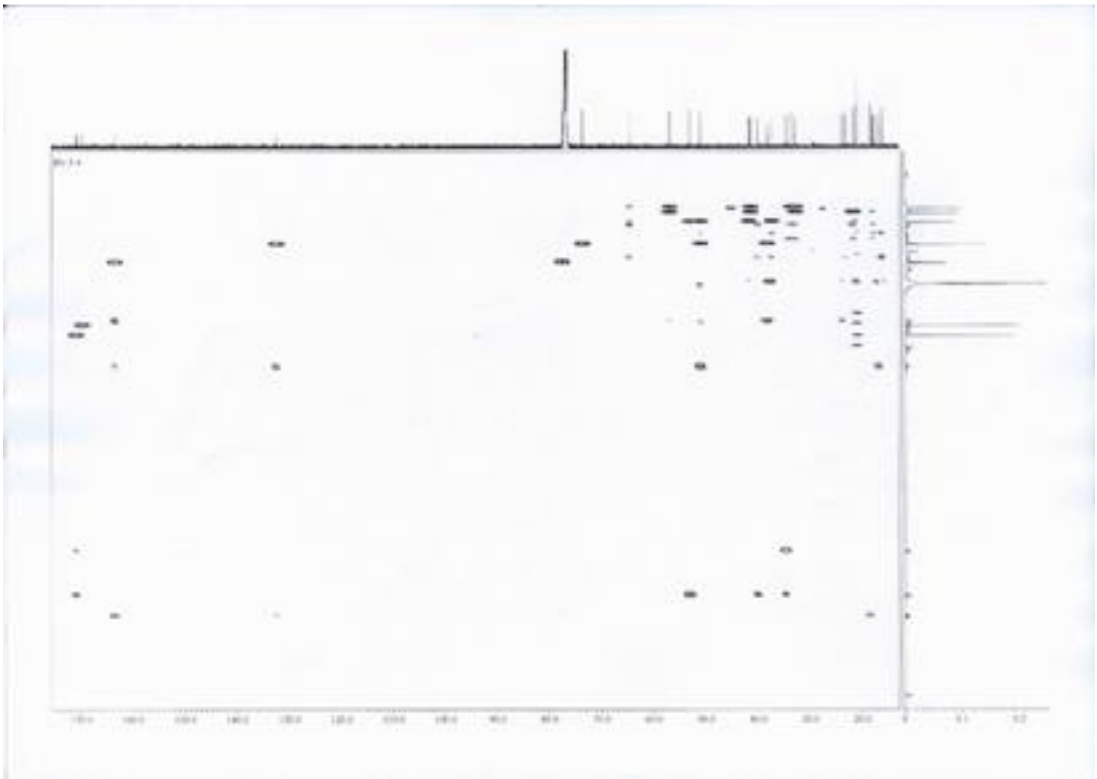

S30. HMBC spectrum of compound **4** in $CDCl_3$.

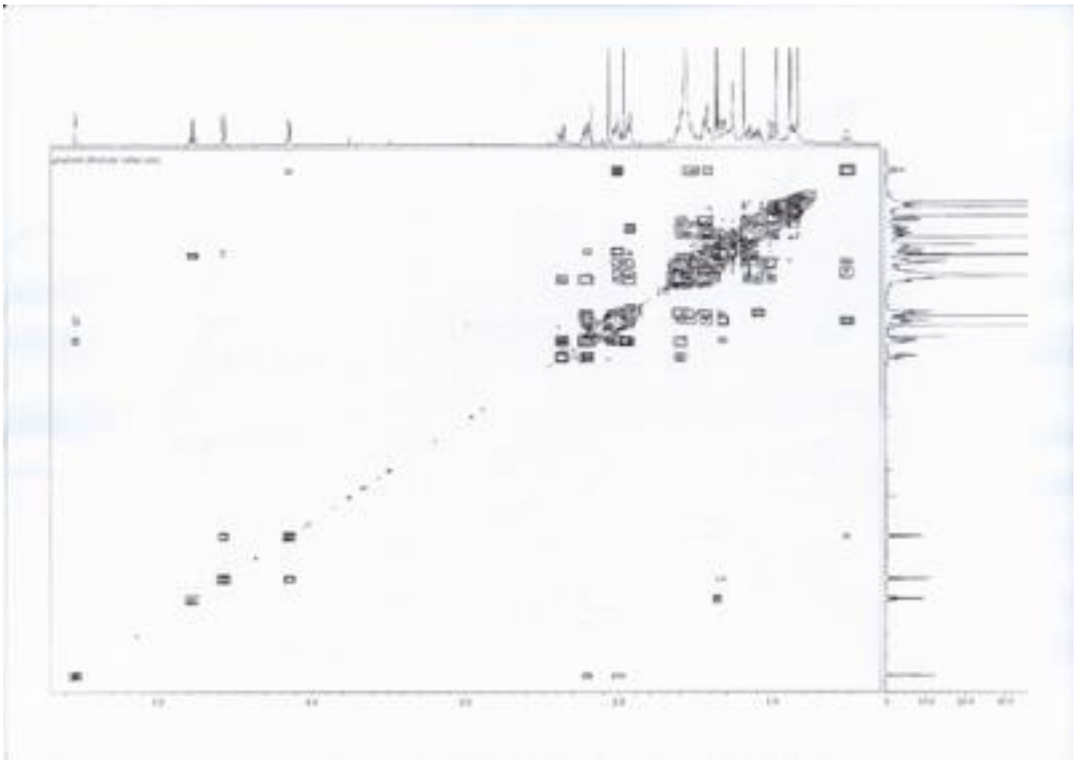

S31. ^1H-^1H COSY spectrum of compound **4** in $CDCl_3$.

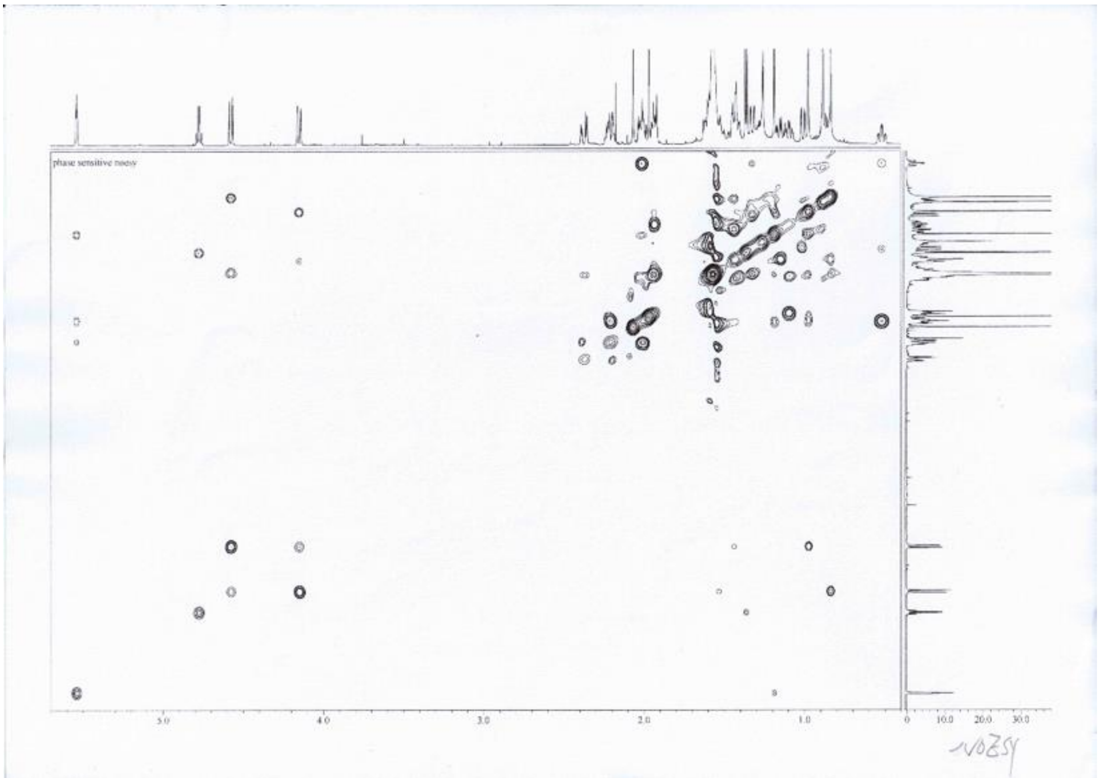

S32. NOESY spectrum of compound **4** in $CDCl_3$
